# Large discrepancies in dominant microphysical processes governing mixed-phase clouds across climate models

**DOI:** 10.1038/s41612-026-01342-7

**Published:** 2026-02-10

**Authors:** Hannah C. Frostenberg, Montserrat Costa-Surós, Paraskevi Georgakaki, Ulrike Proske, Georgia Sotiropoulou, Eleanor May, David Neubauer, Patrick Eriksson, María Gonçalves Ageitos, Athanasios Nenes, Carlos Pérez García-Pando, Øyvind Seland, Luisa Ickes

**Affiliations:** 1 Department of Space, Earth and Environment, Chalmers University of Technology, Gothenburg, Sweden; 2 Barcelona Supercomputing Center, Barcelona, Spain; 3 Laboratory of Atmospheric Processes and their Impacts (LAPI), School of Architecture, Civil & Environmental Engineering, École Polytechnique Fédérale de Lausanne, Lausanne, Switzerland; 4 Institute for Atmospheric and Climate Science, ETH Zurich, Zurich, Switzerland; 5 Center for the Studies of Air Quality and Climate Change, Institute of Chemical Engineering Sciences, Foundation for Research and Technology Hellas, Patras, Greece; 6 Department of Project and Construction Engineering, Universitat Politècnica de Catalunya, Terrassa, Spain; 7 Catalan Institution for Research and Advanced Studies, Barcelona, Spain; 8 Norwegian Meteorological Institute, Oslo, Norway; 9 Present Address: Leipzig Institute for Meteorology, Leipzig University, Leipzig, Germany; 10 Present Address: Hydrology and Environmental Hydraulics, Wageningen University, Wageningen, the Netherlands; 11 Present Address: Department of Physics, National and Kapodistrian University of Athens, Athens, Greece; 12 Present Address: GeoSphere Austria, Vienna, Austria

**Keywords:** Climate sciences, Planetary science

## Abstract

The balance between liquid and ice in clouds remains a major challenge in climate modeling, largely due to uncertainties in ice-related processes. We investigate the relative importance of four microphysical processes—primary ice nucleation (PIN), secondary ice production (SIP), sedimentation, and transport of ice crystals—for the supercooled liquid fraction (SLF) in mixed-phase clouds using three global climate models: EC-Earth3-AerChem, NorESM2-MM, and ECHAM6.3-HAM2.3. All models identify PIN as the dominant influence on SLF at cold temperatures in high northern latitudes, but diverge elsewhere and for higher temperatures. Implementing a unified SIP parameterization produced varied model responses, revealing fundamental differences in how microphysical processes interact within each model framework. These discrepancies suggest that each model prioritizes different processes in shaping the cloud phase. Such divergence may limit the reliability of conclusions regarding microphysical processes drawn from any single model.

## Introduction

The relative amounts of liquid and ice play a crucial role in the climatic impacts of clouds (e.g., Matus and L’Ecuyer^1^). In mixed-phase clouds (MPCs), this balance is commonly quantified by the supercooled liquid fraction (SLF), defined as the ratio of liquid water content to the sum of total condensate (liquid plus cloud ice water content). SLF is often underestimated by global climate models (e.g., Komurcu et al.^2^), leading to biases that can artificially reduce estimates of equilibrium climate sensitivity^3,4^. This implies that, due to inaccuracies in simulating SLF, Earth’s true climate sensitivity may be higher than currently projected by climate models.

In climate models, cloud phase partitioning—and thus SLF—is influenced not only by dynamics but also by various microphysical processes. Ideally, these processes should influence SLF consistently in all global climate models (GCMs). To test this, we analyze simulations from three GCMs, focusing on key microphysical processes. The process initially forming ice crystals in MPCs, where temperatures range from approximately −38 °C to 0 °C, is primary ice nucleation (PIN), also known as “heterogeneous freezing”. This process requires the presence of ice-nucleating particles (INPs) to facilitate the transition from liquid or vapor to ice crystals. Once ice formation is initiated, the number and mass of ice crystals in warmer MPCs (−25 °C ≲ *T* ≤ 0 °C) can be substantially enhanced by secondary ice production (SIP), which includes mechanisms such as rime splintering (Hallett-Mossop^5^ process), collisional break-up of frozen hydrometeors (e.g., Takahashi et al.^6^), and droplet shattering during raindrop freezing (e.g., Griggs and Choularton^7^). SIP appears crucial to reconcile observed ice crystal number concentrations with observed INP concentrations^8–10^. By increasing the number of ice crystals, SIP enhances cloud glaciation, which can significantly alter cloud properties and precipitation processes. The associated release of latent heat further modifies the temperature distribution in the cloud. However, SIP remains either poorly represented or entirely absent in most climate models. Other processes influencing the amount and size of ice crystals include transport (e.g., entrainment, advection, and diffusion of particles) and sedimentation. Sedimentation both seeds lower clouds with ice crystals from aloft via the seeder-feeder mechanism^11^ and removes ice from clouds, which can dehydrate the local atmosphere. This, in turn, can suppress SIP activity. Conversely, the smaller ice crystals produced by SIP tend to sediment more slowly, which may reduce precipitation efficiency. Additionally, the Wegener–Bergeron–Findeisen process (WBF) redistributes mass between liquid droplets and ice crystals, depending on the vapor pressure and the size of the hydrometeors. When the vapor pressure is above ice saturation but below water saturation, ice crystals grow at the expense of evaporating liquid droplets, enhancing ice mass while reducing liquid mass. The collection processes of all hydrometeors in MPCs can lead to larger hydrometeors, which are more prone to sedimentation or precipitation. Their increased size can also influence water attachment and detachment rates. Collectively, these processes impact the phase balance and thus SLF.

We now turn to previously established insights into the process-dependence of SLF. PIN is not its sole determinant—other processes play equally important roles in determining cloud phase^2^. In their study, Komurcu et al.^2^ replaced the existing PIN parameterizations in several models with a unified formulation. Although this improved SLF in some models, it did not reduce the inter-model spread, indicating that other processes significantly affect cloud phase partitioning. They emphasized the need to investigate a broader range of processes to better understand and eventually reduce uncertainties in cloud phase representation.

Building on this, the main goal of our study is to analyze the relative importance of PIN, SIP, sedimentation, and transport of ice crystals on modeled SLF using three GCMs: EC-Earth3-AerChem, NorESM2-MM, and ECHAM6.3-HAM2.3. The models have different microphysical schemes and resolutions (see "Methods"), but this is within the limits of, e.g., the CMIP6 ensemble^12^. Due to technical constraints, we were unable to isolate WBF in some models and, therefore, excluded it from our process analysis. Nevertheless, the statistical method applied in this study effectively captures the relative contributions of the four included processes by comparing them to each other. A central question is to what extent there is a consistent hierarchy of process importance across all three models at varying temperatures.

Additionally, we investigate the parameterization of SIP. In all three models, we implemented the RaFSIP scheme^13^, a machine-learning-based parameterization that accounts for the Hallett-Mossop process, ice-ice collisional break-up, and droplet shattering. The unified implementation allows us to assess the impact of a consistent SIP parameterization across all models.

To evaluate model behavior, we first examine differences in the simulated SLF among the three models and in comparison with satellite observations.

## Results

We analyze the SLF simulated by three GCMs: EC-Earth3-AerChem, NorESM2, and ECHAM6-HAM (see “Methods” and Sections S1–S3 for model descriptions). All results are based on monthly averages for the year 2018. We accumulated the data in 2 °C bins, spaced every 5 °C, between −5 °C and −35 °C. A given bin, centered at temperature *T*_0_, contains the average of all model grid cells in the troposphere where the temperature *T* is *T*_0_ − 1 °C < *T* < *T*_0_ + 1 °C and where liquid and/or cloud ice water content is larger than the Fortran epsilon for doubles (2.220446 ⋅ 10^−16^ kg m^−3^). We begin our analysis by comparing SLF across the three models and against satellite observations. Next, we isolate the impact of a consistent SIP scheme in all models. We conclude by assessing the relative contributions of four key processes to SLF in each model.

### SLF differences across models and their comparison to satellite observations

**Fig. 1:**
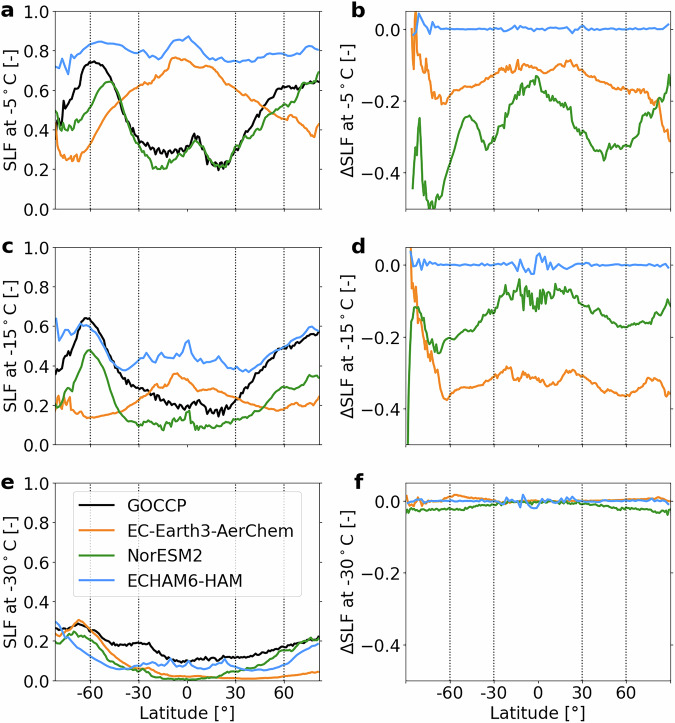
Annual zonal mean SLF for satellite observations and different model configurations. First column: Annual zonal mean SLF for GOCCP and the three models at (**a**) *T* = −5 °C, (**c**) *T* = −15 °C, and (**e**) *T* = −30 °C. Simulations use the unified SIP parameterization. Second column: Difference between simulations with SIP enabled and disabled, for (**b**) *T* = −5 °C, (**d**) *T* = − 15 °C, and (**f**) *T* = −30 °C. Vertical dashed lines indicate the latitudinal zones used in Fig. 3.

To evaluate how well the three GCMs simulate SLF, we first compare the predicted annual and zonal means of SLF (using the unified SIP parameterization RaFSIP, see “Methods” and Section S5) amongst models and with satellite retrievals (left column of Fig. 1). The satellite dataset used is GOCCP (GCM-Oriented CALIPSO, Cloud-Aerosol Lidar and Infrared Pathfinder Satellite Observation, Cloud Product^14^, see "Methods" for details). A major limitation in comparing the models with these satellite data lies in the inconsistent SLF definition: GOCCP derives SLF from the number of pixels classified as containing either liquid or ice, resulting in a frequency-based metric. In contrast, the models calculate SLF based on water contents, i.e., using a mass-based definition. Additional limitations of GOCCP include varying sensitivity to different hydrometeor sizes, constraints on the optical depth range of detectable clouds, and a coarse 16-day revisit time. Despite these limitations, GOCCP remains the most suitable observational dataset for evaluating cloud partitioning, particularly since it is temperature-resolved.

Using GOCCP as a reference, we examine the performance of each model (see also Root Mean Square Error (RMSE) in Table S1). EC-Earth3-AerChem exhibits a latitudinal distribution that differs remarkably from the other models and GOCCP. Specifically, for *T* ≥ −15 °C, EC-Earth3-AerChem simulates the highest SLF in the tropics and the lowest at high latitudes (Fig. 1a, c). In contrast, the other models and GOCCP show the opposite pattern: SLF is minimal in the tropics and maximal at higher latitudes. These discrepancies are probably due to EC-Earth3-AerChem overestimating supercooled liquid water in the tropics and ice at higher latitudes and for the warmest MPC temperatures, while at −15 °C it underestimates supercooled liquid water at higher latitudes (see also the water contents in Fig. S3; note that SLF non-linearities limit direct water content comparisons, see Section S6). However, at *T* = −25 °C, the SLF simulated by EC-Earth3-AerChem agrees remarkably well with GOCCP (Fig. S2 and Table S1). At even lower temperatures, the SLF simulated by EC-Earth3-AerChem at southern high latitudes continues to match that of GOCCP, while EC-Earth3-AerChem underestimates supercooled liquid at other locations (Fig. 1e and Table S1). The low SLF at high northern latitudes and low temperatures is a clear indication of overly efficient heterogeneous freezing in the model. The other biases in EC-Earth3-AerChem may be linked to known limitations or inconsistencies in its saturation adjustment and convective detrainment scheme that are extensively discussed in Costa-Suróos et al.^15^ and summarized in Section S1. They lead to a negative SLF bias throughout the vertical column at higher latitudes and around cloud tops in the mid- and high latitudes, as well as a positive SLF bias in the tropics.

NorESM2 matches GOCCP in the tropics and lower mid-latitudes at *T* = −5 °C (Fig. 1a). However, at lower temperatures, it considerably underestimates SLF (Fig. 1c, e and Table S1). Although NorESM2 probably overestimates ice (see cloud ice water content in Fig. S3), its latitudinal SLF distributions agree qualitatively with those of GOCCP. At *T* = −30 °C and below, the bias decreases at higher northern latitudes and in the Southern Hemisphere at colder temperatures, although notable discrepancies persist in the tropics (Fig. 1e and Table S1).

At *T* = −5 °C, ECHAM6-HAM overestimates supercooled liquid water and underestimates cloud ice, especially in the tropics, compared to GOCCP and the other models (Fig. 1a, Fig. S3 and Table S1). At *T* = −15 °C, the SLF simulated by ECHAM6-HAM matches GOCCP in the mid- and high latitudes, but remains too high in the tropics (Fig. 1c and Table S1). The zonal distribution differs considerably from the satellite observations, with too high SLF in the tropics at higher temperatures. At *T* = −30 °C, ECHAM6-HAM underestimates SLF compared to GOCCP, but has the smallest bias in the tropics (Fig. 1e and Table S1).

In summary, all models display notably different latitudinal distributions of SLF, and although there is some agreement in simulated SLF for specific regions and temperatures, there is no universal agreement among the models or with observations. These deviations indicate significant differences in how microphysical processes are represented across the models, underscoring the need to investigate the factors driving these patterns.

### Model-specific responses to a unified SIP parameterization

Building on the SLF discrepancies between the models and GOCCP, we now assess the impact of SIP by comparing simulations with and without a unified SIP parameterization. Note that the model simulations presented in the previous section included SIP. We implemented the RaFSIP parameterization across all models (see “Methods” and Section S5; note that the models were not retuned after adding RaFSIP). This uniform SIP formulation provides a consistent framework for comparing model responses to a single microphysical process.

Introducing SIP leads to a direct mass transfer from liquid to cloud ice through the Hallett-Mossop and droplet shattering processes, thereby decreasing SLF. Ice-ice collisional breakup, on the other hand, initially increases only the ice crystal number concentration, but enhanced WBF rates^16,17^ are expected to reduce SLF as well. Consistent with expectations, EC-Earth3-AerChem and NorESM2 exhibit a substantial SLF decrease in the temperature range where SIP is active in all three models (i.e., *T* ≥ −20 °C, Fig. 1b, d). In EC-Earth3-AerChem, ∣ΔSLF∣ is very similar in the tropics and mid-latitudes, preserving its characteristic zonal SLF pattern (middle column in Fig. S2). This means that including SIP brings EC-Earth3-AerChem closer to GOCCP in the tropics, while the bias increases in the mid- and high latitudes. A notable feature in EC-Earth3-AerChem is the minimal ∣ΔSLF∣ in the southern high latitudes at *T* = −15 °C (Fig. 1d), likely due to pre-existing low SLF and consequently limited liquid water at the South pole (see Fig. S2), a prerequisite for some SIP mechanisms. The temperature at which the largest ∣ΔSLF∣ occurs differs across models: for EC-Earth3-AerChem, it peaks at *T* = −15 °C, while for NorESM2, the largest change occurs at *T* = −5 °C and diminishes with colder temperatures (Fig. 1b, d). In NorESM2, the strongest SIP effects are found in regions with high SLF, i.e., SIP slightly evens out the zonal differences in its SLF (middle column in Fig. S2). This stronger effect at high SLF suggests an interaction with WBF that is very efficient in this model, potentially converting excessive liquid water to ice. At *T* = −5 °C, SIP brings the SLF simulated by NorESM2 into close agreement with GOCCP in the mid-latitudes and the tropics (Fig. 1a and middle column in Fig. S2). However, at lower temperatures, it causes SLF to fall below observational estimates (Fig. 1c).

In contrast to EC-Earth3-AerChem and NorESM2, ECHAM6-HAM shows virtually no change in SLF when SIP is activated (Fig. 1b, d, f), consistent with its known insensitivity to SIP reported in Proske et al.^18^.

In summary, while all models incorporate the same SIP parameterization, their responses vary significantly in both magnitude and spatial distribution. This highlights the significant impact of model-specific characteristics on microphysical process interactions.

### Different processes dominate SLF variability across models

**Fig. 2:**
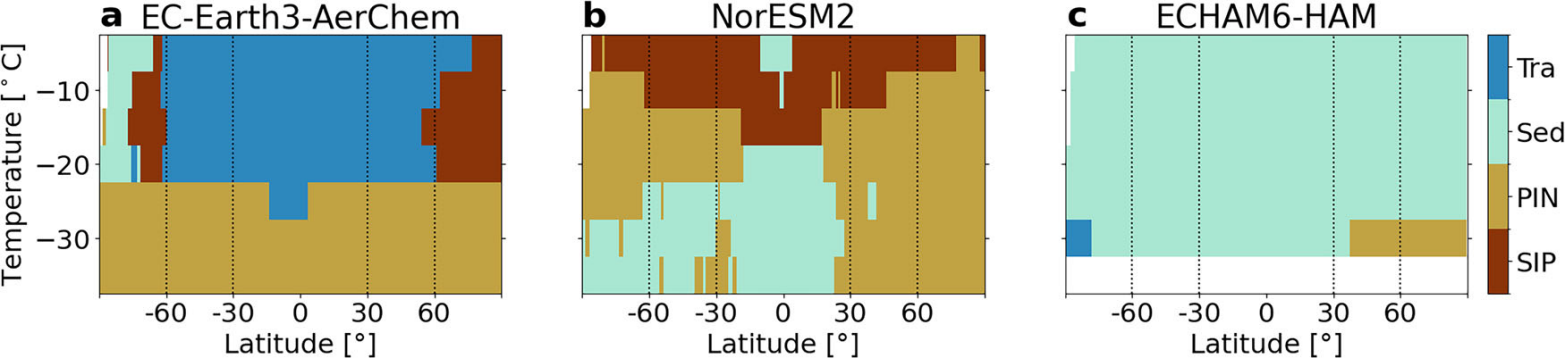
Dominant process contributing to the variability of zonal and annual mean SLF. **a** EC-Earth3-AerChem, **b** NorESM2, **c** ECHAM6-HAM. Vertical dashed lines indicate the latitudinal zones used in Fig. 3.

**Fig. 3:**
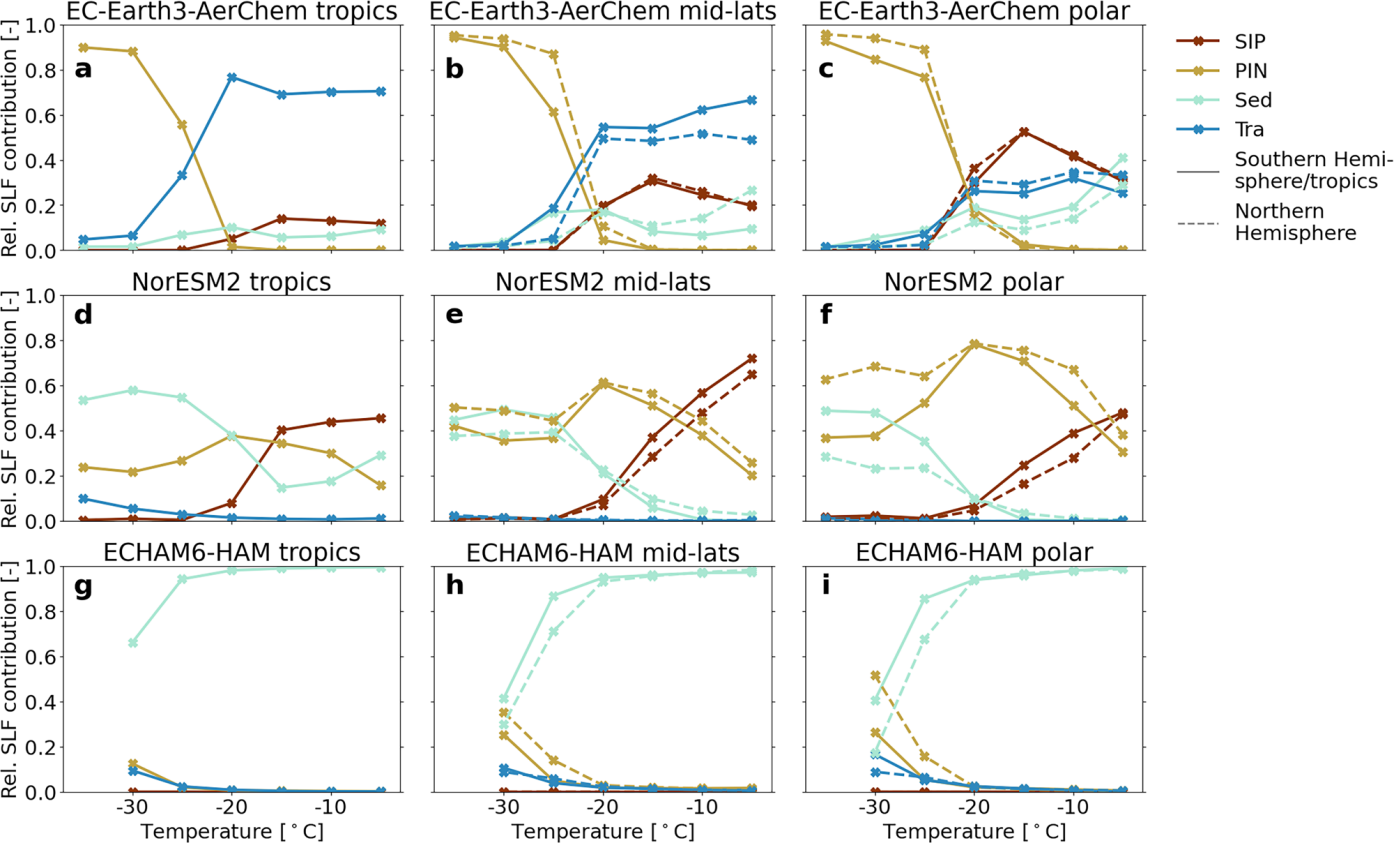
Relative contributions of four processes to the variability of annually and spatially averaged SLF. **a**–**c** EC-Earth3-AerChem, **d**–**f** NorESM2, **g**–**i** ECHAM6-HAM. Tropics: 30^∘^ S-30^∘^ N (**a**, **d**, **g**), mid-latitudes: 30–60^∘^ S/N (**b**, **e**, **h**), polar: 60–90^∘^ S/N (**c**, **f**, **i**, see vertical dashed lines in Figs. 1 and 2 for latitudinal boundaries).

To further investigate the factors influencing modeled SLF and the distinct responses to a unified SIP parameterization across models, we analyze the relative importance of four key processes: SIP, PIN, sedimentation (Sed), and transport (Tra) of ice crystals. An important aspect is that Tra includes detrainment from convective clouds in EC-Earth3-AerChem and NorESM2, but not in ECHAM6-HAM. To quantify the relative importance of the four processes, we use the factorial method. This approach requires simulations with all combinations of the four processes turned on or off, resulting in 16 simulations per model (see “Methods” for more details). For each model, Fig. 2 shows the dominant process for SLF variability by latitude and temperature, whereas Fig. 3 presents the relative contribution of the four processes. Note that for ECHAM6-HAM, we excluded *T* = −35 °C as it considers all clouds at *T* ≤ −35 °C to be ice clouds, while this threshold lies at *T* ≈ −38 °C for the other models (see Table 1).

**Table 1:** Summary of the resolution and relevant microphysics information for each model

Model	EC-Earth3-AerChem	NorESM2	ECHAM6-HAM
Model version (atmospheric component)	EC-Earth3-AerChem-3.3.4.1 (IFS Cy36r4)	NorESM2-MM (CAM6-OsloAero5.3)	ECHAM6.3-HAM2.3 (ECHAM6.3)
Main reference	van Noije et al.^41^	Seland et al.^42^	Tegen et al.^43^
Resolution	T255, approx. 0.7° lat × 0.7° lon, 91 levels, 2700 s	T127, approx. 1° lat × 1° lon, 32 levels, 600 s	T63, approx. 1.9° lat × 1.9° lon, 47 levels, 450 s
Microphysics scheme	Single-moment, Forbes et al.^44^	Double-moment, Gettelman and Morrison^45^	Double-moment, Lohmann et al.^46^
PIN representation	Aerosol-aware, immersion freezing by K-feldspar, quartz, and marine organic aerosol	Aerosol-aware, classical nucleation theory-based immersion, contact, and deposition freezing by dust and black carbon aerosol	Aerosol-aware, immersion and contact freezing by montmorillonite and immersion freezing by black carbon aerosol
PIN references	Costa-Surós et al.^15^, Thomas et al.^47^, Harrison et al.^48^, Wilson et al.^49^	Hoose et al.^50^, Kirkevåg et al.^51^, Wang et al.^52^	Hoose et al.^53^, Lohmann and Diehl^54^
Cirrus threshold (Temperature threshold between mixed-phase and cirrus regime, *T*≤*x*: all ice clouds)	−38 °C	≈ −38 °C, but also depends on sub-grid updraft velocity, humidity, and sulfate and coarse mode dust number concentrations^55^	−35 °C
Sedimentation velocity of ice crystals *v*_*i*_	*v*_*i*_ = 0.15 m/s and adjustment for temperature and pressure variations^56^	*v*_*i*_ = 700/s ⋅ *D*_*i*_ with *D*_*i*_ ice crystal diameter (in m)^57^. With limit of ${D}_{i,\max }=350\,{\rm{\mu }}$m: ${v}_{i,\max }=0.245$ m/s	Sedimentation velocity depending on ice crystal radius, atmospheric temperature and pressure, and a height correction factor^58^
WBF representation	Not explicitly represented. Implicitly included through the saturation adjustment scheme^44^ (see also SectionS1).	Flux from liquid droplets to ice crystals, depending, i.a., on ice and liquid saturation mixing ratios^57^	When *w* < w^*^, liquid water will be forced to evaporate and be deposited onto the existing ice crystals within that timestep, and saturation with respect to ice is assumed^59,60^ (see also Section S3)
Phase partitioning in convective clouds at MPC temperatures	Ice fraction depends on temperature as smooth hyperbolic function centered around −6.7 °C such that at *T* ≲ −23 °C nearly all convective condensate is treated as ice^61,62^	Mainly, ice fraction is 1 if *T* < −35 °C and 0 if *T* > − 5 °C with linear dependency on temperature in between	Between 0 °C and −35 °C, convective clouds are considered all liquid if *w* > w^*^ and all ice if *w* < w^*^
Saturation adjustment (with respect to phase partitioning)	Handled in several parts of the model (including turbulent mixing, first-guess cloud, the cloud scheme, and semi-Lagrangian trajectory averaging at the end of the time step) with different assumptions^63^ (see also Section S1).	Supersaturation always relaxed by condensation to cloud water	If *w* > w^*^, saturation with respect to water assumed and excess vapor condenses on cloud droplets, if *w* < w^*^ saturation with respect to ice assumed and excess vapor deposits on ice crystals

At almost all latitudes and temperatures, the models disagree on which process has the largest relative impact on SLF (Fig. 2). Consistent agreement across all models is found only at high northern latitudes and the coldest temperatures (right bottom corners), where SLF variability is dominated by PIN. This model agreement strongly suggests that PIN is a key process affecting SLF in the Northern Hemisphere at low temperatures. The local dominance of PIN can be explained by higher dust emissions in the Northern Hemisphere, driven by its larger land area, and the PIN parameterizations in the models, all of which heavily depend on dust INPs (see Table 1). At all other latitudes and temperatures, our analysis based on the three models does not allow us to identify a single dominant process for SLF, as the key process varies between models. From our previous SLF evaluation, one might conclude that agreement between a model and GOCCP at specific latitudes and temperatures could help constrain the relative importance of the four processes. However, two key considerations complicate this interpretation. First, like all observational datasets, GOCCP carries uncertainties. Unfortunately, these have not been quantified, so it remains unclear in which regions the models agree with GOCCP. Second, even if a model does reproduce the zonal SLF pattern observed by GOCCP, it may still exhibit a systematic bias (e.g., NorESM2 at −15 °C, Fig. 1c). In such cases, the dominant process identified by the factorial method could be responsible for the bias, suggesting that it exerts an unrealistically strong influence on SLF and should instead rank as the second-most important contributor. Therefore, agreement in zonal SLF patterns alone does not imply that the model accurately represents the relative importance of the contributing processes, since there could be compensating errors.

A closer look at the dominant processes reveals that ECHAM6-HAM stands out, with Sed dominating SLF across nearly all latitudes and temperatures (Fig. 2c). It is essential to point out that sedimentation in this version of ECHAM6-HAM is artificially increased to prevent cloud cover and the radiative effect of cirrus becoming too large^19^. In contrast, EC-Earth3-AerChem and NorESM2 agree that PIN dominates at mid- to high latitudes in the Northern Hemisphere at *T* ≤ −25 °C, and that SIP dominates at some higher latitudes at *T* = −5 °C. However, the overall latitudes and temperatures where SIP dominates SLF differ remarkably between EC-Earth3-AerChem and NorESM2, despite using the same parameterization. Sed dominates at different latitudes and temperatures for EC-Earth3-AerChem and NorESM2. Note that in NorESM2, the apparent importance of Sed might be underestimated due to necessary code modifications (see Section S2). Tra is the major contributor to SLF variability in EC-Earth3-AerChem in the tropics and mid-latitudes at higher temperatures. Given this, and as previously discussed, SLF biases in EC-Earth3-AerChem in both the tropics and at higher latitudes may largely stem from detrainment. This is consistent with known deficiencies in its phase partitioning during convective detrainment. Since horizontal advection and vertical diffusion are unlikely to dominate the Tra term, detrainment is the most plausible driver of this bias. In contrast to EC-Earth3-AerChem, Tra has a negligible influence in NorESM2 and only a localized effect in ECHAM6-HAM. This limited effect in ECHAM6-HAM might be caused by its Tra term not including detrainment from convective clouds. Notably, although EC-Earth3-AerChem and NorESM2 include the same subprocesses in their Tra terms, its importance differs markedly between the two models.

In summary, except for agreement on PIN at low temperatures in the higher northern latitudes, the models show no consistent pattern in dominant process behavior. This discrepancy limits our ability to infer the true atmospheric controls on SLF from model intercomparison alone.

### Different relative importance of all processes across models

Examining the complete process contributions (Fig. 3), we again observe substantial inter-model variability, reinforcing the lack of consensus in identifying their dominant process. Consistent with previous results, SIP is important in EC-Earth3-AerChem and NorESM2 (especially in Fig. 3c, e), but negligible in ECHAM6-HAM (Fig. 3g–i). However, the temperature and latitude where SIP is most important differ between EC-Earth3-AerChem and NorESM2, despite the use of the same RaFSIP parameterization. All models agree that PIN is more important towards the poles, especially in the Northern Hemisphere (Fig. 3c, f, i), as already seen and discussed in the previous section. In EC-Earth3-AerChem, SLF at *T* ≤ −30 °C is influenced exclusively by PIN, whose relevance rapidly diminishes with increasing temperature and becomes negligible by *T* ≥ −20 °C (Fig. 3a–c). Despite the low SLF values simulated by EC-Earth3-AerChem at *T* ≤ −30 °C in Fig. 1e, the spread across all simulations is considerable (Fig. S11 first row), underscoring the robustness of the process analysis. The pronounced influence of PIN at the coldest MPC temperatures indicates that these clouds are highly sensitive to inaccuracies in the PIN parameterization. This sensitivity likely helps explain the EC-Earth3-AerChem bias relative to satellite observations (Fig. 1e). Unlike EC-Earth3-AerChem, NorESM2 shows a peak in the relative importance of PIN at *T* = −20 °C, with values remaining relatively uniform across all temperatures. ECHAM6-HAM shows PIN peaking at the lowest temperature in the northern polar region (Fig. 3i). The influence of Sed on SLF varies considerably between the models. Sed is consistently the least important process for EC-Earth3-AerChem (Fig. 3a–c). Its relatively low ice crystal sedimentation velocity (see Table 1) likely explains why the SLF simulated by EC-Earth3-AerChem is less sensitive to Sed than in the other models. Slower sedimentation increases the likelihood that an ice crystal sublimates before reaching lower-level MPCs. In contrast to EC-Earth3-AerChem, Sed is the most important process in NorESM2 at low temperatures and in the tropics with decreasing importance toward the poles (Fig. 3d–f), although the previously discussed code modifications may artificially reduce its importance. On the other hand, SLF variability between NorESM2 simulations is small for low temperatures, especially in the tropics (Fig. S11 middle row), so the process analysis is less robust here. For ECHAM6-HAM, Sed is the dominant process, as already shown (Fig. 2c). Figures 3g-i emphasize that for *T* ≥ −20 °C, processes other than Sed have a negligible impact on SLF. Similar to PIN at colder temperatures in EC-Earth3-AerChem, this suggests that clouds at warmer MPC temperatures in ECHAM6-HAM are highly sensitive to Sed parameterization uncertainties—likely the result of the aforementioned artificially enhanced sedimentation in ECHAM6-HAM, which may also explain its insensitivity to SIP. In general and for all models, Sed is probably tied to freezing in higher levels where all clouds are purely ice clouds via the seeder-feeder process^11^. Focusing on the last process, Tra is the most important process for EC-Earth3-AerChem at *T* ≥ −20 ^∘^C in the low and mid-latitudes (Fig. 3a, b), as Fig. 2a showed. As discussed previously, known limitations in the convective detrainment in EC-Earth3-AerChem, a component of its Tra term, may contribute to the large SLF bias in the tropics at warm temperatures. The pronounced importance of Tra may also indicate that other processes contribute less than they should in EC-Earth3-AerChem, especially compared to NorESM2, where several processes are relevant at almost all locations and temperatures. In contrast to EC-Earth3-AerChem, the Tra contribution to SLF in NorESM2 is minimal (Fig. 3d–f). It has a small effect on SLF in ECHAM6-HAM at very low temperatures in all regions (Fig. 3g–i). As noted previously, the Tra term in ECHAM6-HAM does not include detrainment from convective clouds. If it did, Tra might have been more important, potentially reducing the apparent importance of Sed.

For NorESM2 and ECHAM6-HAM, there appears to be an interrelation between Sed and PIN when comparing both hemispheres at low temperatures (Fig. 3e, f, h, i): Sed and PIN show hemispheric differences in SLF relevance of similar magnitude. This suggests that the presence of potent INPs in the Northern Hemisphere diminishes the importance of the sedimentation of ice crystals. At higher temperatures in NorESM2, SIP, rather than Sed, seems to compensate for PIN in the Southern Hemisphere compared to the Northern Hemisphere, especially in the polar region (Fig. 3f). In NorESM2 and ECHAM6-HAM, one process—Tra in NorESM2 and SIP in ECHAM6-HAM—is almost irrelevant to SLF at all temperatures and locations, while all processes are relevant at some temperatures and locations in EC-Earth3-AerChem.

Finally, the order in which microphysical processes are called differs among models, but this sequence does not appear to influence the relative importance of each process for SLF. Thus, the general structural and parametric differences between models are more likely to account for the observed divergences.

In summary, our analysis reveals that apart from consistent PIN dominance at low temperatures in the Northern Hemisphere, there is no inter-model agreement on the most or least important process controlling SLF. These results emphasize the complexity of modeling mixed-phase clouds and point to the need for improved understanding and parameterization of microphysical processes in climate models.

## Discussion

Comparing the zonal annual means of SLF simulated by EC-Earth3-AerChem, NorESM2, and ECHAM6-HAM revealed considerable discrepancies, particularly in the zonal patterns at warmer temperatures. While individual models agree with the satellite observations in some specific regions and temperatures, the overall comparison suggests notable deviations between models and observations. Implementing the unified SIP parameterization RaFSIP in all three models enabled a direct comparison between SLF simulated with and without the identical SIP parameterization, revealing model-dependent impacts on both magnitude and spatial pattern. In ECHAM6-HAM, SLF remained virtually unchanged, while EC-Earth3-AerChem and NorESM2 showed marked, but differing, reductions in SLF. In particular, the inclusion of SIP did not systematically reduce biases relative to the observations, underscoring that parameterization improvements do not guarantee better alignment with satellite data. Our process analysis further revealed the underlying causes of these differences in modeled SLF: the relative contributions of four processes that are potential sources of ice crystals in MPCs (SIP, PIN, sedimentation, and transport of ice crystals) vary substantially across models, with little consensus across temperatures or latitudes. The only consistent finding was that all three models identified PIN as the dominant process influencing SLF at the coldest temperatures in the higher northern latitudes. At other locations and temperatures, however, the models diverged in both the dominant processes and their relative contributions to SLF variability. Our findings underscore that robust conclusions about process importance in real mixed-phase clouds cannot be drawn from a single climate model. However, process analyses for individual models can provide insights into their internal behavior and help to identify inconsistencies.

Our study does not offer a solution to the long-standing issue that GCMs perform poorly in simulating SLF, as highlighted by Komurcu et al.^2^. Had our models demonstrated consistent process-dependence, we could have given recommendations to observationalists and modelers about which processes are most critical to understand better and represent more accurately in the models—or at least which processes are less impactful and require less focus.

Limitations of our analysis include the absence of convective detrainment in the ECHAM6-HAM transport term and the omission of the WBF process from the analysis due to technical constraints in two models. Analyzing models where WBF can be isolated, alongside other GCMs with this capability, could provide valuable insights into the importance of WBF for SLF in MPCs. Generally, broadening the analysis to include more models that differ significantly in their design (see the model genealogy proposed by Kuma et al.^20^) could yield more robust insights into the relative importance of the different processes for the cloud phase in MPCs. Since MPCs vary considerably depending on geographic location and season, narrowing the analysis to a specific region and time may help to infer at least regional and seasonal process relevance. For example, a study focusing on the Southern Ocean is already planned, where model results at a higher temporal resolution will be compared to observations from the SOCRATES campaign. Such targeted studies, anchored in local high-quality observations, may help identify which models most accurately simulate SLF in specific regions and seasons, and clarify the reasons for their better performance. Beyond the four microphysical processes analyzed here, accurate cloud simulation in GCMs is also limited by coarse model resolution and inadequate representation of the multiscale nature of clouds, including liquid-ice inhomogeneity within clouds (e.g., Chylek and Borel^21^), and mesoscale cloud organization (e.g., Vial et al.^22^). Incorporating these multiscale aspects may yield improvements that potentially could be comparable to those from refining microphysical parameterizations.

The disagreement of the models on the importance of the different processes for SLF highlights the need to analyze microphysical processes directly, i.e., as process rates, rather than relying solely on state variables^23,24^. Observational constraints on these processes—ideally studied collectively—would be valuable for improving model representation. Both observational campaigns and model development efforts need to take a comprehensive approach that accounts for the collective influence of all relevant processes.

While improving the representation of the microphysical processes is a logical next step, our results raise a fundamental question: Is it reasonable to represent the intricate and poorly constrained system of cloud microphysics through deterministic parameterizations of individual physical processes? Although we do not explore this philosophical question in depth, acknowledging it provides important context for two complementary directions for future model improvement. The first is to refine observational constraints in order to improve the representation of all microphysical processes simultaneously, rather than individually. Alternatively, cloud microphysics could be represented through a unified statistical approach that captures the net effect of these processes, based on large-scale state variables (in line with, e.g., Berner et al.^25^ and Lovejoy^26^). Such developments could reduce uncertainties in cloud phase simulations and, ultimately, help constrain Earth’s climate sensitivity.

In summary, our study demonstrates that the relative importance of four key microphysical processes for cloud phase varies substantially across three GCMs. These findings highlight the need for targeted observational efforts and offer guidance for future model development strategies.

## Methods

### Statistical framework for process analysis: factorial method

The factorial method enables the estimation of the mean sensitivity of the outcome variable, in this case SLF, to each factor, in this case, the four processes. In addition, it allows for the analysis of interactions between factors, in which the effect of one factor may depend on the state of another. However, we excluded interactions from the analysis since we found that they are much lower than the effects of the individual processes (see Fig. S10). We used the results of the factorial method and applied analysis of variance (ANOVA). ANOVA identifies how much of the total variability in SLF can be explained by each factor and their interactions. It does not take into account which sign the effect has on SLF; rather, only the absolute amount is considered. Our study examines four factors (the four processes) at two levels (active or inactive) without replication, resulting in a 2^4^ experimental design. This approach entails 16 simulations for each of the three models, covering all possible combinations of the four processes turned either on or off (see Table S3). For detailed descriptions of the method, see Montgomery^27^, Teller and Levin^28^, or Ickes et al.^29^.

To perform the calculations, we used the Python package statsmodels v. 0.14.2^30^. Prior to analysis, SLF was averaged annually, zonally, and across latitudinal zones (for Fig. 3) using cdo^31^.

### Overview of climate model simulations

Table 1 summarizes, for each model, the resolution and model characteristics that are relevant to this study. For more detailed descriptions of the models, see Sections S1–S3.

All simulations share the same configuration, differing only in which selected processes are turned on or off (see Table S2). The simulation configuration follows the requirements for AeroCom phase III experiments (https://aerocom.met.no/experiments, last access: 28 August 2025): The simulations cover the year 2018 with a spin-up period of three months (i.e., 1 October to 31 December 2017). Anthropogenic aerosol and precursor emissions, as well as biomass-burning emissions, are taken from the Community Emission Data System (CEDS) or CMIP6 scenarios. For volcanic emissions, the dataset for AeroCom by S. Carn is used. Sea surface temperature (SST) and sea ice are prescribed by different datasets for each model. Horizontal winds (or vorticity and divergence) and surface pressure are nudged towards reanalysis data for 2018 from ERA-5 or ERA-Interim. The configuration options for specific models are listed in Sections S1–S3.

### Satellite observations of SLF: GOCCP

The GCM-Oriented CALIPSO (Cloud-Aerosol Lidar and Infrared Pathfinder Satellite Observation) Cloud Product (GOCCP)^14^ is a dataset designed for the evaluation of cloud properties in GCMs. GOCCP derives cloud properties from Level-1 measurements by the Cloud and Aerosol Lidar with Orthogonal Polarization (CALIOP) lidar—a two-wavelength lidar operating at wavelengths of 532 and 1064 nm, mounted on the CALIPSO satellite. CALIPSO ancillary data include temperatures from GMAO (Global Modeling and Assimilation Office) based on GEOS^32^ (Goddard Earth Observing System). The CALIOP Level-1 data have a vertical resolution of 30–60 m. In the GOCCP product, these data are interpolated onto a grid with a vertical resolution of 480 m, which is comparable to the resolution of our models (see Fig. S1). Cloud detection is performed on each pixel of the lidar profile, using the native 30–60 m resolution. The phase of each cloudy pixel is determined by evaluating the change in the polarization state of the backscattered signal, as nonspherical ice hydrometeors cause a larger polarization change^33^. Pixels at temperatures *T* > 0 °C are always classified as liquid, while those at *T* < −42 °C are classified as ice. The liquid cloud fraction is calculated by dividing the number of liquid-classified pixels by the total number of pixels within a given latitude-longitude grid box.

Challenges in model-satellite comparisons arise from differences in sensor sensitivity to hydrometeor sizes and cloud layers, as well as varying definitions of variables such as cloudiness^34^. It is therefore important to note that the CALIOP lidar is more sensitive to smaller hydrometeors in mixed-phase clouds, such as supercooled liquid droplets, than to larger hydrometeors, such as ice crystals. Furthermore, the lidar signal is fully attenuated for clouds with an optical thickness greater than approximately 3^35^. An additional limitation of the phase classification by GOCCP is that, in cases of multi-layered or optically thick clouds, cloudy pixels at lower altitudes are excluded^33^. The phase classification is also performed only above a minimum optical thickness of around 0.03^14^, where this exact threshold depends on cloud thickness and particle size. This implies that very optically thin clouds may not be included in the aggregation of phase data.

Model-satellite comparisons are typically statistical in nature, due to the relatively low temporal resolution of polar-orbiting satellites at a given location—every 16 days in the case of CALIPSO—and differing cloud formations simulated by the models at a given time point. The aim of our analysis is to compare annually and zonally averaged SLF at every 5 °C within a temperature range of −5 to −35 °C. GOCCP provides the relative fraction of ice in a cloud with respect to the total amount of condensate, *CF*_ice/liq_ calculated as the ratio between the number of ice pixels and the total number of pixels. For our purposes, we take the SLF as equal to 1 − *CF*_ice/liq_. However, for the models, the SLF is calculated as the ratio of liquid water content to the sum of liquid and cloud ice water content. Although comparing a ratio of pixel numbers (GOCCP) to a ratio of mass (models) is not ideal, GOCCP remains the most suitable observational dataset for our purposes. GOCCP data are available as monthly means, pre-binned into temperature bins of 3 °C. To ensure consistency with the models, we interpolated GOCCP SLF onto 1 °C bins. We then averaged annually and over 2 °C wide bins such that a bin centered at a given temperature *T* contains the average SLF within *T* −1 °C < *T* < *T* + 1 °C. Any differences between the original data and the interpolated data were found to be small. We did not adjust for the differing vertical resolutions between GOCCP and the models, since there is no combined GOCCP dataset available including SLF, temperature, and altitude simultaneously. An estimate of potential errors caused by vertical resolution differences showed a negligible impact on the temperature levels analyzed (see Section S4 and Fig. S4).

### Unified SIP parameterization RaFSIP

The SIP representation in our models follows the second version of the Random Forest SIP (RaFSIP) scheme, developed by Georgakaki and Nenes^13^. RaFSIP is a data-driven parameterization that uses machine learning on mesoscale simulation outputs with explicit SIP microphysics. The training dataset for RaFSIP was taken from 2-year regional climate simulations (2016–2017) with the mesoscale Weather Research and Forecasting (WRF) model. The simulations focused on polar stratiform clouds over the pan-Arctic region. Cloud microphysics was parameterized using the double-moment scheme of Morrison et al.^36,37^, which includes the Hallett-Mossop rime splintering (HM) mechanism^38^. The scheme was augmented to include ice-ice collisional break-up (BR) and droplet-shattering (DS) based on Phillips et al.^39,40^. RaFSIP was trained by applying a Random Forest Regressor (RFR) algorithm to a subset of the model output. See Georgakaki and Nenes^13^ for more details on the formulation of the parameterization and Section S5 for technical details of the implementation into the three models used in this study. RaFSIP was implemented to operate at temperatures between −20 ≤ *T* ≤ 0 °C, where the impact of SIP is most pronounced. Depending on *T* and the presence of rime on ice hydrometeors (RIMR, mass tendency of raindrops collected by ice crystals), we expect different SIP processes to be efficient, thereby activating different RFRs. These are summarized in Table 2, with the suffix of each RFR name denoting the SIP processes, and ALL referring to HM, BR, and DS being active. One difference between the RaFSIP implementations in the models is that for ECHAM6-HAM and NorESM2, all used hydrometeor variables are in-cloud values, whereas EC-Earth3-AerChem uses grid-box-averaged values. Comparing two EC-Earth3-AerChem simulations—one using in-cloud values and one using grid-box-averaged values—showed only minor differences, which are unlikely to affect our study.

**Table 2:** Summary of the different RFRs activated under different microphysical and thermodynamic conditions

RFR	Conditions		RaFSIP predictions				
	Temperature	RIMR	BR rate	HM rate	DS rate	*Qc*_*tr*_	*Qr*_*tr*_
forestBRwarm	−3 ≤ *T* ≤ 0 °C	-	*✓*	✗	✗	✗	✗
forestALL	−8 < *T* < − 3 °C	>0	*✓*	*✓*	*✓*	*✓*	*✓*
forestBRHM	−8 < *T* < − 3 °C	=0	*✓*	*✓*	✗	*✓*	✗
forestBRDS	−20 ≤ *T* ≤ − 8 °C	>0	*✓*	✗	*✓*	✗	*✓*
forestBR	−20 ≤ *T* ≤ − 8 °C	=0	*✓*	✗	✗	✗	✗

For ECHAM6-HAM, the mass mixing ratio of raindrops (*Qr*) is used instead of RIMR, see Section S5. *Qc*_*tr*_: transferred mass tendencies of cloud droplets, *Qr*_*tr*_: transferred mass tendencies of raindrops.

## Supplementary information

Supplementary information

## Data Availability

The datasets generated and analyzed during the current study are available at 10.5281/zenodo.17266270. GOCCP data can be downloaded from https://climserv.ipsl.polytechnique.fr/cfmip-obs/Calipso_goccp.html (last access: 4 October 2025).

## Code availability

The EC-Earth model is restricted to institutes that have signed a memorandum of understanding or letter of intent with the EC-Earth consortium and a software license agreement with the ECMWF. Confidential access to the code and the data used to produce the simulations described in this paper can be granted to editors and reviewers; please use the contact form at http://www.ec-earth.org/about/contact to obtain the code or contact the corresponding author directly. NorESM is open-source and freely available at https://github.com/NorESMhub/NorESM. See https://noresm-docs.readthedocs.io/en/latest/(last access: 4 August 2025) for its documentation. The NorESM code version used to produce the results presented here is available at https://github.com/geosoti/NorESM/tree/v2.1-forces-npj-Frostenbergetal2026. The ECHAM6-HAMMOZ model is freely available to the scientific community under the HAMMOZ Software License Agreement, which defines the conditions under which the model can be used. The specific version of the code used for this study is archived in the ECHAM6-HAMMOZ SVN repository at https://svn.iac.ethz.ch/external/ECHAM6-HAMmoz/echam6-hammoz/tags/papers/2025/Frostenberg_et_al_2025_NPJ_Clim_Atmos_Sci. More information can be found on the HAMMOZ website (https://redmine.hammoz.ethz.ch/projects/hammoz, last access: 13 June 2025).
