# Supplementary material for: Large discrepancies in dominant microphysical processes governing mixed-phase clouds across climate models
Source: NPJ Clim Atmos Sci. 2026 Feb 10;9(1):75. doi: 10.1038/s41612-026-01342-7 (PMC13021502; doi:10.1038/s41612-026-01342-7)
Supplement: Supplementary file 1 — Supplementary information [file 41612_2026_1342_MOESM1_ESM.pdf]

Supplementary Information to "Large discrepancies in dominant microphysical processes governing mixed-phase clouds across climate models"

Hannah C. Frostenberg[1*], Montserrat Costa-Surós[2], Paraskevi Georgakaki[3,4], Ulrike Proske[5,6], Georgia Sotiropoulou[7,8], Eleanor May[1], David Neubauer[5,9], Patrick Eriksson[1], María Gonçalves Ageitos[2,10], Athanasios Nenes[3,7], Carlos Pérez García-Pando[2,11], Øyvind Seland[12], Luisa Ickes[1*]

[1]Department of Space, Earth and Environment, Chalmers University of Technology, 41296 Gothenburg, Sweden.
[2]Barcelona Supercomputing Center, 08034, Barcelona, Spain.
[3]Laboratory of Atmospheric Processes and their Impacts, École Polytechnique Fédérale de Lausanne, 1015, Lausanne, Switzerland.
[4]Current address: Leipzig Institute for Meteorology, Leipzig University, 04103, Leipzig, Germany.
[5]Institute for Atmospheric and Climate Science, ETH Zurich, 8092, Zurich, Switzerland.
[6]Current address: Hydrology and Environmental Hydraulics, Wageningen University, 6708 PB, Wageningen, the Netherlands.
[7]Center for the Study of Air Quality and Climate Change, Foundation for Research and Technology Hellas, 70013, Patras, Greece.
[8]Current address: National and Kapodistrian University of Athens, 15772, Athens, Greece.
[9]Current address: GeoSphere Austria, 1190, Vienna, Austria.
[10]Department of Project and Construction Engineering, Universitat Politècnica de Catalunya, 08222, Terrassa, Spain.
[11]Catalan Institution for Research and Advanced Studies, 08010, Barcelona, Spain.
[12]Norwegian Meteorological Institute, 0313, Oslo, Norway.

*Corresponding author(s). E-mail(s): hannah.frostenberg@chalmers.se; luisa.ickes@chalmers.se;

Supplementary Texts

S1 Model description: EC-Earth3-AerChem

The simulations were run with the EC-Earth3-AerChem version 3.3.4.1 of the EC-Earth model [1]. In this configuration, the Integrated Forecasting System (IFS) from the European Centre for Medium-Range Weather Forecasts (ECMWF) serves as the atmospheric module of EC-Earth3-AerChem. Its horizontal resolution was set to approximately 0.7°. Additionally, the EC-Earth3-AerChem model was coupled to the 3-D chemistry Tracer Model 5 TM5 [2]. TM5 features 34 vertical levels and a horizontal resolution of 3° latitude by 2° longitude. The coupling between EC-Earth3-AerChem and TM5 occurred every 6 model hours.

The anthropogenic aerosol and precursor emissions follow the CMIP6 scenario SSP2-4.5. For biomass-burning emissions, the CEDS data are being used. SST and sea ice are prescribed by PCMDIv1.1.8. Wind vorticity and divergence in the upper levels of the atmosphere are nudged towards ERA-5.

EC-Earth3-AerChem uses the IFS version Cycle 36r4 (Cy36r4). This version employs a single-moment cloud microphysics scheme [3]. The ice microphysics scheme of IFS Cy36r4 is based on two processes: homogeneous ice crystal nucleation and ice crystal growth by deposition in mixed-phase clouds, while the heterogeneous freezing in this study follows the aerosol- and temperature-sensitive parameterization by Costa-Surós et al. [4]. It is based on the immersion freezing of K-feldspar and quartz [5], and of marine organic aerosol [6]. CCN (cloud condensation nuclei) activation follows [7–10]. The WBF process is not explicitly represented as a distinct microphysical process in IFS Cy36r4. Instead, it is implicitly included through the saturation adjustment scheme. When a model grid cell contains both liquid and ice and is supersaturated with respect to ice but subsaturated with respect to liquid, water vapor preferentially deposits onto ice crystals. This leads to the growth of cloud ice and the reduction of supercooled liquid water. However, this treatment is diagnostic and instantaneous, with no prognostic representation of mixed-phase evolution. As a result, the WBF process is included only implicitly via equilibrium thermodynamics, and is not parameterized as an explicit physical process with its own rate equations. Consequently, it cannot be isolated or directly modified without changes to the core saturation adjustment logic in the source code. In IFS Cy36r4, saturation adjustment is handled

in several parts of the model (including turbulent mixing, first-guess cloud, cloud formation, and semi-Lagrangian trajectory averaging at the end of the time step). Each of these components may apply different assumptions, particularly the turbulence scheme, which diverges from the rest of the model. This fragmented structure makes it difficult to trace interactions between processes and complicates efforts to isolate or modify specific mechanisms, such as the WBF process, without inadvertently affecting the core saturation adjustment logic [11].

Limitations of EC-Earth3-AerChem: The atmospheric component of EC-Earth3-AerChem, IFS Cycle 36r4, has known limitations or inconsistencies that are extensively discussed in Costa-Surós et al. [4]. In summary, inconsistencies in cloud saturation adjustments within the mixed-phase regime (0°C to −38°C) lead to a bias toward ice crystal formation over liquid droplets in mid- and high latitudes, reducing supercooled liquid water at cloud tops and contributing to shortwave radiation biases (R. Forbes, personal communication). Additionally, while the stratiform cloud scheme included physically based mixed-phase processes, the convective detrainment scheme retained a simple temperature-dependent diagnostic approach with all condensate treated as ice when the temperature is $\lesssim -23$°C. This approach may contribute to positive biases in SLF in the tropics and negative biases at higher latitudes. These biases are independent from the recent introduction of aerosol aware ice-nucleation and secondary ice production, as reflected in results using the standard IFS Meyers temperature-based ice nucleation (Fig. S2). Subsequent updates in IFS allowed convective clouds to retain liquid condensate below 600 hPa at relatively low temperatures, increasing the SLF in convective cold-air outbreaks, and reducing radiation biases in key regions like the Southern Ocean, North Atlantic, and North Pacific [12].

S2 Model description: NorESM2

For the simulations we used a NorESM2-MM version similar to Seland et al. [13], but with an improved representation of mixed-phase clouds [14] and a horizontal resolution of approximately 1°. The atmospheric component of NorESM2 is CAM6-Oslo, which consists of the Community Atmosphere Model version 6 (CAM6) and the OsloAero5.3 aerosol scheme [15].

Unlike the other models, NorESM2 simulations have a spin-up time of 4 months, from 1 September 2017 to 31 December 2017. Anthropogenic aerosol and precursor emissions follow the CMIP6 scenario SSP2-4.5. The 2015 historic global biomass burning emissions for CMIP6 (BB4CMIP) based on merging satellite observations with proxies and fire models [16] are used for biomass-burning emissions. Volcanic

emissions are represented by the CMIP6 stratospheric aerosol forcing based on the Global Space-based Stratospheric Aerosol Climatology [17], also for 2015. SST and sea ice are based on the SST and ICE boundary dataset created from merged Reynolds/HADISST products, as in Hurrell et al. [18]. Horizontal wind and surface pressure are nudged towards ERA-Interim.

CAM6-Oslo employs the Gettelman and Morrison [19] double-moment microphysics scheme. Heterogeneous PIN parameterizations follow the Classical Nucleation Theory (CNT) which accounts for immersion, contact, and deposition freezing of cloud droplets, depending on the surface areas and contact angles of cloud-borne dust and black carbon particles. The CNT formulation is based on Hoose et al. [20] with further improvements using a probability density function of contact angles [21]. A correction of the contact angle model has recently been applied by Kirkevåg et al. [15]. CCN activation follows Abdul-Razzak and Ghan [8].

Model adaptations in NorESM2: As described in Methods in the main manuscript, individual processes were disabled in the 16 simulations required for the factorial method. In NorESM2 simulations where sedimentation was turned off, we observed a significant accumulation of IWC, leading to unrealistic model behavior. ICNC did not increase proportionally, leading to unusually large average ice crystal diameters. To address this issue, we modified the autoconversion routine by Gettelman and Morrison [19]. Specifically, in grid boxes where the in-cloud mean cloud ice diameter exceeds 350 μm, all cloud ice (of all sizes, both mass and number) is transferred to the snow category. This adjustment ensures that snow, which is still subject to sedimentation, helps mitigate the accumulation of ice mass. However, this modification artificially reduces the impact of sedimenting ice crystals in our NorESM2 simulations, likely leading to an underestimation of the importance of sedimentation in this model. This limitation is evident in the simulation where all four processes were disabled: ice produced during the model spin-up remained in the atmosphere indefinitely rather than being removed.

S3 Model description: ECHAM6-HAM

The simulations were carried out with ECHAM6.3-HAM2.3, i.e., version 6.3 of the atmospheric general circulation model ECHAM [22] coupled with version 2.3 of the tropospheric aerosol model HAM [23–25] at approximately 1.9° horizontal resolution. The exact model version is based on the one used in Proske et al. [26], with the additional SLF diagnostics and RaFSIP implementation, a COSP update, and AeroCom diagnostics.

Anthropogenic aerosol and precursor emissions are taken from CEDS, biomass-burning emissions from the CMIP6 scenario SSP3-7.0. SST and sea ice data are based on monthly means over 2000-2015, from PCMDI-AMIP 1.1.2. These are merged SST based on HadISST by the UK MetOffice and OI2 by NCEP. Divergence, vorticity, and pressure are nudged towards ERA-Interim.

Cloud microphysics is implemented as a double-moment scheme according to Lohmann et al. [27], Lohmann and Hoose [28], Neubauer et al. [29]. Contact, condensation and immersion freezing are calculated explicitly, taking into account the chemical composition of the IN. Freezing is described as a function of the number of INP, temperature, and cloud liquid water mass-mixing ratio in the cloudy part of the grid box [30, 31]. The concentration of INP and CCN is interactively calculated in the model. Aerosol activation in warm clouds is described by Abdul-Razzak et al. [7] and Abdul-Razzak and Ghan [8] (see Tegen et al. [25], Stier [32], Lohmann and Neubauer [33]). The WBF process is represented as a threshold process depending on vertical velocity (w) [34]: when w exceeds a threshold vertical velocity w^*, both ice crystals and cloud droplets coexist. When $w < w^*$, liquid water will be forced to evaporate and be deposited onto the existing ice crystals within that timestep and saturation with respect to ice is assumed. w^* depends on supersaturation with respect to liquid water and ice, ice crystal number concentration and radius, and a coefficient depending on temperature and pressure [28].

S4 Vertical resolution of GOCCP and the models

The GOCCP data are gridded at 480 m vertical resolution, designed to be comparable to climate model vertical spacing. However, in the first Results section of the main manuscript, we compare the models to GOCCP at specific temperature bins. Since no GOCCP product provides both temperature and altitude simultaneously, direct remapping to the vertical levels of the models was not possible.

To assess the potential impact of vertical resolution differences on model-satellite SLF comparison, we interpolated the altitude-binned GOCCP SLF data onto the vertical grid of each model (Fig. S4a-d) and calculated the SLF zonal mean within narrow height layers (Fig. S4e-l). Since the SLF data remains identical, any differences between zonal means arise solely from the vertical grid. These differences are generally small, except around approx. 5 km in altitude (Fig. S4f, g, j). At this height, GOCCP SLF exhibits a sharp vertical gradient in the tropics, and thus the interpolated results are more sensitive to how the vertical layers align with this transition. The effect is most pronounced for models with coarser vertical grids (ECHAM and NorESM), whose

vertical levels lead to particularly thick layers in the tropics. At higher altitudes and outside the tropics, the differences are minimal. However, GOCCP values at the original resolution near 5 km altitude in the tropics (dashed black lines in Fig. S4f, g, j) are considerably higher than the tropical GOCCP values shown in Fig. 1a. This indicates that the tropical GOCCP values in Fig. 1a correspond to altitudes above 5 km, likely near 6 km, where vertical resolution differences introduce negligible SLF errors (Fig. S4h, k). We therefore conclude that vertical resolution differences do not affect our model-satellite comparison in the first Results section of the main manuscript.

S5 Implementation of the secondary ice production parameterization RaFSIP

The modular nature of the RaFSIP scheme allows its seamless integration within the microphysics routine of our three models, provided that the input features are well located inside the stratiform cloud microphysics routine. The workflow is described in Fig. S5 and discussed below. The different SIP processes are represented by different Random Forest Regressors (RFRs, see Table 2 in the main manuscript). All RFRs take the fields of temperature (in K), relative humidity with respect to ice (unitless), liquid water content (LWC in kg kg^{-1}), cloud ice water content (IWC in kg kg^{-1}), and the mass tendency of cloud droplets rimed onto ice crystals (RIMC in kg kg^{-1} s^{-1}) as inputs to predict ice production rates for each SIP mechanism. Additionally, the mass tendency of rain drops rimed onto ice crystals (RIMR in kg kg^{-1} s^{-1}) is employed as input for RFRs considering the effect of DS (i.e., forestALL and forestBRDS). These six inputs balance computational simplicity with physical relevance, enabling the scheme to approximate complex SIP parameterizations (e.g., Phillips et al. [35, 36]), while capturing the key conditions for SIP activity. RaFSIP provides the production rates of secondarily formed ice hydrometeors (in kg^{-1} s^{-1}) attributable to each SIP mechanism – namely BR_{rate}, HM_{rate} and DS_{rate}. Furthermore, it provides the transferred mass tendencies of cloud droplets (Qc_{tr} via the HM process) and raindrops (Qr_{tr} via the DS process) to small cloud ice hydrometeors (in kg kg^{-1} s^{-1}) when the corresponding mechanism is active.

LWC and IWC in EC-Earth3-AerChem and NorESM2 consider contributions from all liquid and ice hydrometeors. In ECHAM6-HAM, however, raindrops and snowflakes are excluded due to their sedimentation within a single timestep. Riming rates and IWC were identified as the most important input variables, significantly enhancing the predictive skills of RaFSIP (see Figures S6-S10 in the Supporting Information of Georgakaki and Nenes [37]). NorESM2 incorporates parameterizations for both

RIMC and RIMR, when cloud droplets and raindrops are collected by snow hydrometeors, enabling straightforward RaFSIP integration. In ECHAM6-HAM, with only a RIMC parameterization, adjustments are made to forestALL and forestBRDS to make predictions with five inputs instead of the standard six. The final selection of which RFR to use in the corresponding temperature range is based on the presence of a nonzero mass mixing ratio of raindrops (Qr) at the current timestep, which is the criterion employed in ECHAM6-HAM to identify cases where DS might be taking place (Fig. S5).

While the offline evaluation of all RFRs is thoroughly detailed in Georgakaki and Nenes [37], Figs. S6 and S7 present results for the forestALL and forestBRDS RFRs modified for implementation in ECHAM6-HAM. In this offline evaluation, each RFR underwent testing on the four-month dataset (January, April, July, and October 2017) that was excluded from the training process. The exclusion of RIMR from forestALL (Fig. S6) and forestBRDS (Fig. S7) was identified as primarily influencing the predictions of DS_{rate} and Qr_{tr}. Indeed, the Root Mean Square Error (RMSE) for DS_{rate} and Qr_{tr}, as predicted by RaFSIP, exhibited an increase of up to 40% and 80%, respectively, when compared against the RMSE scores presented in Table 2 of Georgakaki and Nenes [37]. Considering that the impact of each SIP mechanism on the total SIP rate is cumulative and the fact that DS generates significantly smaller rates (up to 3 orders of magnitude) than BR and HM (Figs. S6c and S7b), we argue that the reduced predictive skill of these two RFRs will not substantially affect the overall performance of the RaFSIP scheme in ECHAM6-HAM.

Within EC-Earth3-AerChem, the absence of a parameterization for riming rates hindered the integration of RaFSIP. To overcome this, we developed two additional RFRs, named forestrimall and forestrimc (see the inset of Fig. S5), trained on the same two-year dataset as RaFSIP to diagnose the variables RIMC and RIMR. Utilizing the RandomForestRegressor class from the scikit-learn package version 1.2.0 [38], both RFRs were trained following the same process as RaFSIP, each consisting of 10 decision trees as a compromise between accuracy and computational efficiency. These RFRs use temperature (in K) and mass mixing ratios of relevant liquid and ice species (in kg kg^{-1}) as inputs. In cases where all mass mixing ratios of cloud droplets (Qc), rain (Qr), cloud ice (Qi), and snow (Qs) are nonnegative, forestrimall predicts both RIMC and RIMR. In cases where Qr is zero, forestrimc is employed with the remaining mixing ratios and temperature as inputs to predict RIMC. Predictions from these RFRs are constrained within upper and lower limits of 10^{-6} and 10^{-18} kg kg^{-1} s^{-1}, respectively, corresponding to the bounds observed in the training dataset. The offline performance of forestrimall and forestrimc is provided in Figs. S8 and S9. Note that

the RIMC and RIMR rates do not participate in the conservation equations of EC-Earth3-AerChem, but are used solely for diagnostic purposes in computing inputs for RaFSIP. Once RIMC and/or RIMR are calculated, the workflow in EC-Earth3-AerChem is exactly the same as the one in NorESM2 and ECHAM6-HAM (Fig. S5). Having located the input features in all three models, RaFSIP is called at every model timestep and height where the necessary conditions to activate SIP are met. At the end of each model timestep, the RaFSIP parameterization calculates the total SIP rate (in kg^{-1} s^{-1}) by summing the contributions of the three SIP mechanisms. An upper limit of 10 particles kg^{-1} s^{-1} is imposed, to prevent any explosive SIP behavior that would lead to numerical instabilities in our simulations. Only EC-Earth3-AerChem exhibited sensitivity to this upper limit, which was ultimately treated as a tuning factor, with the limit of 10 kg^{-1} s^{-1} producing more reasonable results compared to the limit of 1 or 100 kg^{-1} s^{-1}. The total SIP rate is then incorporated into the conservation equation for cloud ice number concentration. If HM and/or DS is active, the transported mass Qc_{tr} and/or Qr_{tr} (predicted by RaFSIP) is deducted from the respective liquid category (Qc or Qr) and added to the Qi conservation equation. Similar to the SIP rate, the transferred masses are also constrained, ensuring they do not exceed the total rimed masses RIMC and RIMR.

Technical implementation into the models: To implement RaFSIP in the stratiform cloud microphysics routine of the three GCMs, a Fortran 90 module was developed to read and store the parameters for building all RFRs. Specific ASCII files are exclusively accessed during the initial model timestep, and all RaFSIP parameters are passed as public variables into the corresponding microphysics code. In NorESM2 and ECHAM6-HAM, the HM parameterization included in the default version of the models was deactivated using a switch in the code to avoid double-counting its effect.

S6 Why LWC and IWC averages cannot explain SLF

Figure S3 shows the annual and zonal average liquid and cloud ice water contents (LWC, IWC) in the first two columns. Unfortunately, they cannot always explain SLF as shown in Fig. 1 in the main manuscript (also as third column in Fig. S3). This is caused by the non-linear calculation of SLF (SLF = LWC / (LWC + IWC)). Consequently, computing SLF from annually and zonally averaged LWC and IWC (SLF_calc, fourth column in Fig. S3) does not yield the same result as averaging the SLF values calculated directly from the 3-hourly model output (see fifth column in Fig. S3, showing their absolute difference, and the sixth column showing their relative difference). The solid lines mark locations where the absolute difference is less than 0.1

and the relative difference less than 0.5, indicating that LWC and IWC can reasonably explain SLF.

Despite these differences, certain characteristics remain evident when comparing the models: EC-Earth3-AerChem retains considerably more supercooled liquid water than the other models, particularly in and around the tropics at higher temperatures. Its simulated IWC is consistently larger than in the other models with a similar zonal distribution as NorESM2, but some local maxima in the southern high latitudes. NorESM2 and ECHAM6-HAM simulate very similar LWC, but differing IWC leading to the large discrepancies in SLF. NorESM2 simulates an LWC minimum and IWC maximum in the tropics, which explains why only NorESM2 reproduces the tropical SLF minimum at warmer temperatures observed in GOCCP.

Supplementary Figures

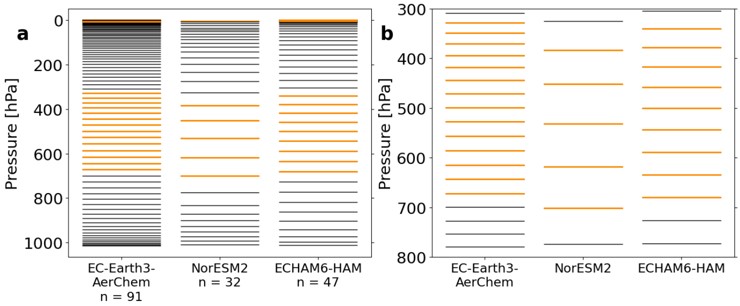

Fig. S1 Annual zonal means of vertical model levels of the three models at 35° S (where the pressure of the lowest model level is ca. 1013 hPa). Orange are levels where the temperature is in the mixed-phase region, i.e., $-38°C < T < 0°C$. (**a**) All levels with linear y-axis, (**b**) close-up of levels in MPC temperature range.

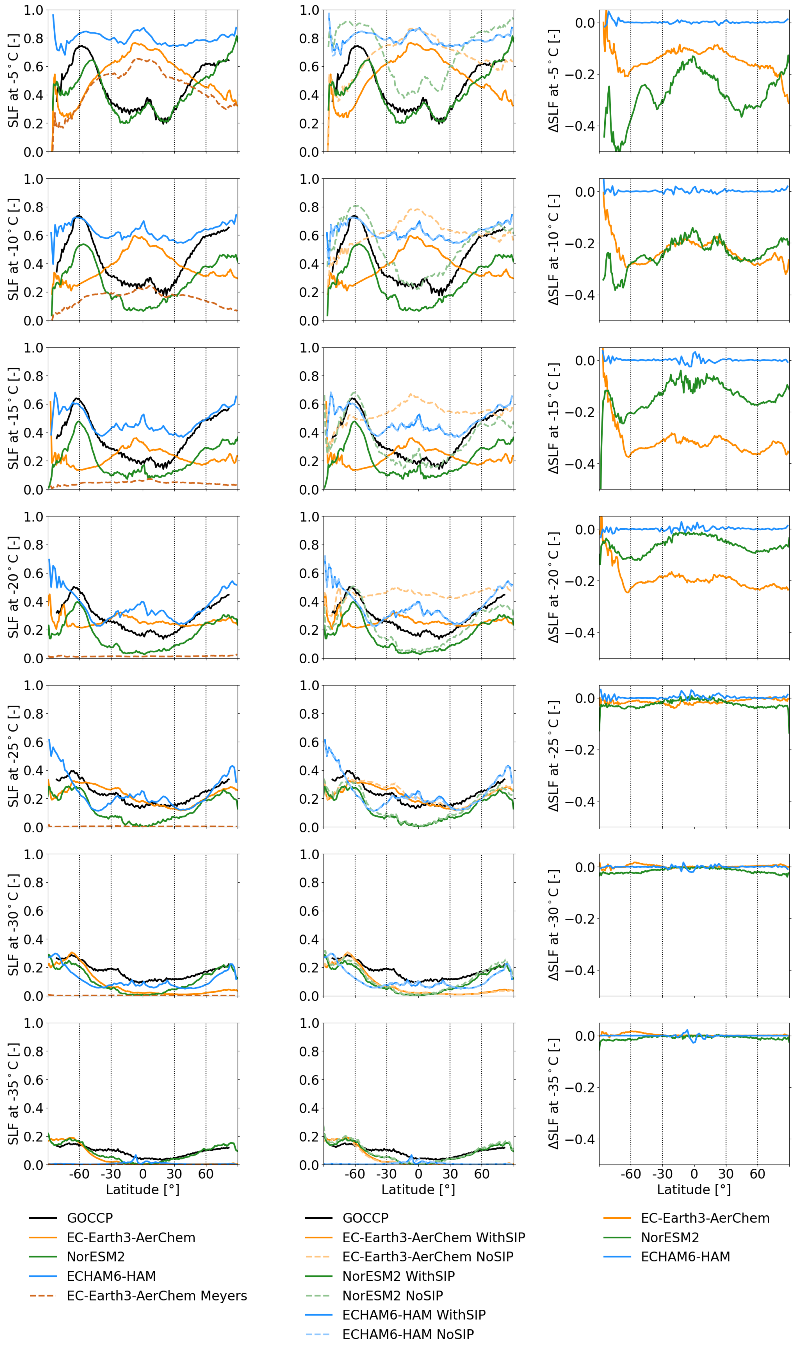

Fig. S2 As Fig. 1 in the main manuscript, but including all temperatures and with the second column showing the annual zonal mean SLF for the three models with (solid lines) and without SIP (dashed lines). EC-Earth3-AerChem Meyers is the default version of EC-Earth3-AerChem with temperature-based PIN following Meyers et al. [39].

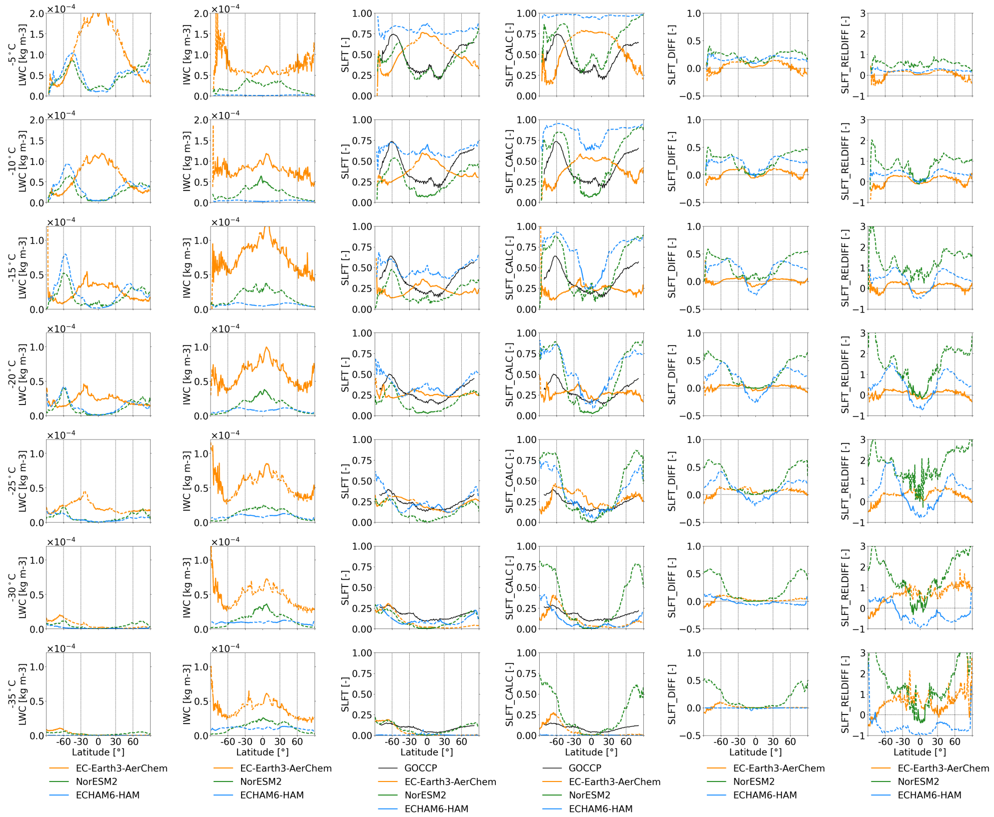

Fig. S3 Annual zonal means of different variables at all MPC temperatures (rows), as in Fig. S2. First column: liquid water content (LWC). Second column: cloud ice water content (IWC). Third column: SLF for GOCCP and the three models (same as first column in Figs. 1 and S2). Fourth column: SLF calculated from the annual zonal mean LWC/IWC in first two column (SLF_CALC). Fifth column: Absolute difference between SLF and SLF_CALC. Sixth column: Relative difference between SLF and SLF_CALC. Only where the lines are solid can the water contents explain SLF, since there the relative difference is less than 0.5 and the absolute difference is less than 0.1.

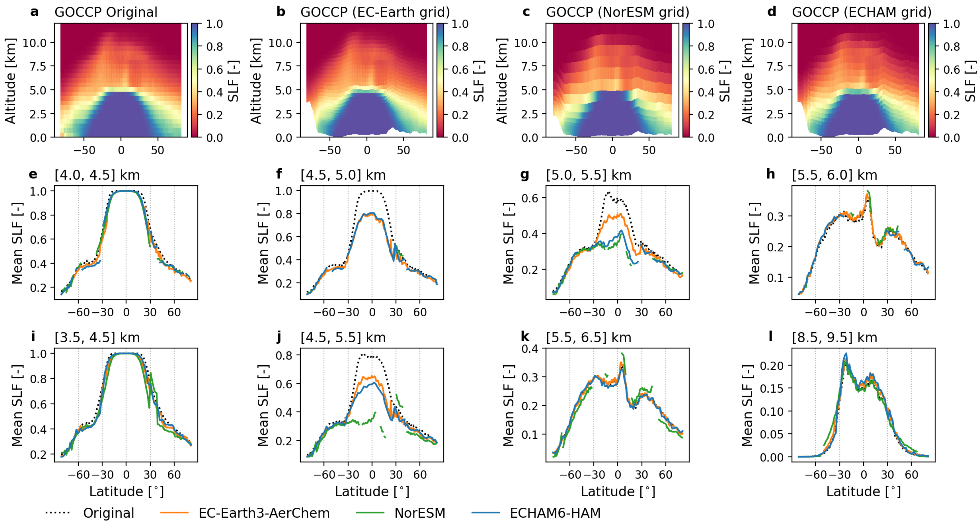

Fig. S4 Sensitivity of annually and zonally averaged GOCCP SLF values to vertical resolution. (**a**) Original GOCCP data on vertical altitude grid, (**b-d**) GOCCP data interpolated on to the annually and zonally averaged model vertical levels of the models, (**e-h**) average SLF within the given altitude range for original GOCCP data, and the GOCCP data interpolated on to each of the model vertical levels. If there is no model level within the given altitude range for a specific latitude, the average SLF is neither calculated nor plotted.

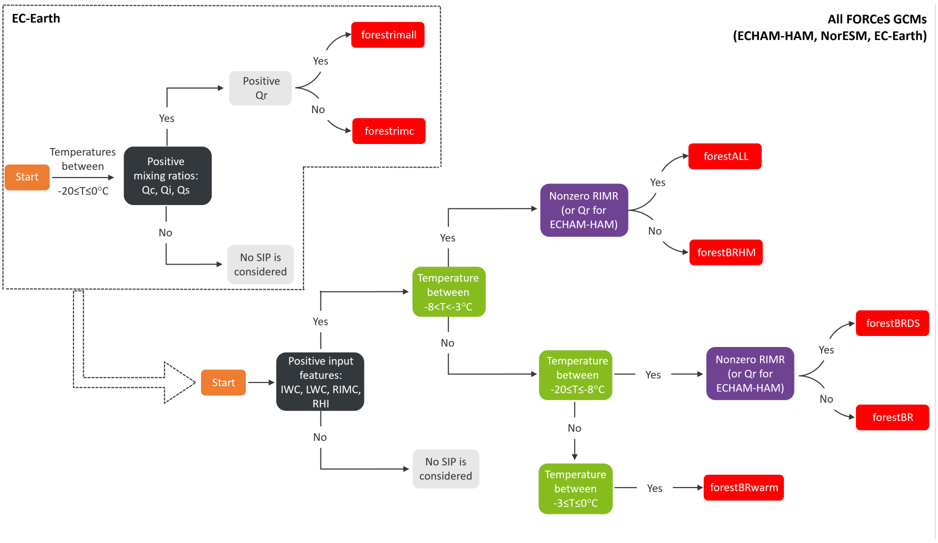

Fig. S5 RaFSIP workflow when implemented into the stratiform microphysics routine of our three GCMs. The red boxes highlight the specific RFRs activated to generate predictions, depending on the meteorological and microphysical conditions at each model timestep. Outputs from these RFRs are presented in Table 2. The inset outlines the EC-Earth3-AerChem workflow, highlighting essential steps for computing the riming rates, RIMC and/or RIMR, subsequently used as inputs to the RaFSIP scheme.

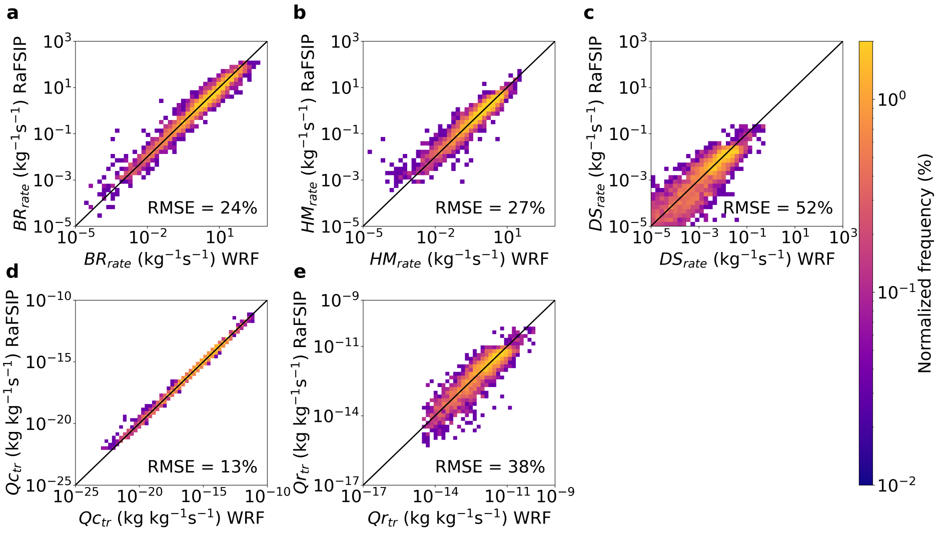

Fig. S6 Normalized histograms (i.e., frequency is scaled by the total number of predictions) of the true WRF results versus the forestALL predictions used in RaFSIP parameterization as implemented into ECHAM6-HAM to predict the (**a**) BR_{rate}, (**b**) HM_{rate}, (**c**) DS_{rate}, and the transferred masses (**d**) Qc_{tr} and (**e**) Qr_{tr}. The black line represents the one-to-one line.

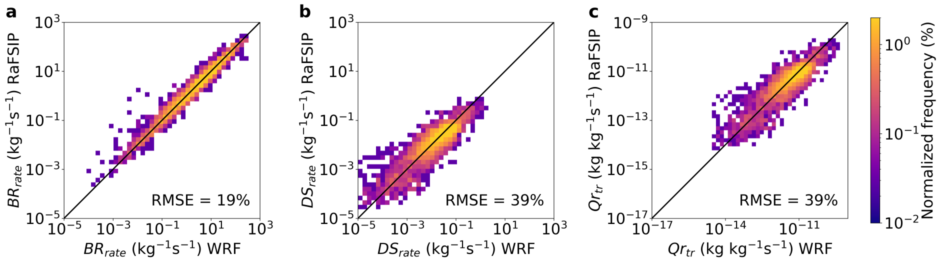

Fig. S7 Normalized histogram of the true WRF results versus the forestBRDS predictions used in RaFSIP parameterization as implemented into ECHAM6-HAM to predict the (**a**) BR_{rate}, (**b**) DS_{rate}, and (**c**) the transferred mass Qr_{tr}. The black line represents the one-to-one line.

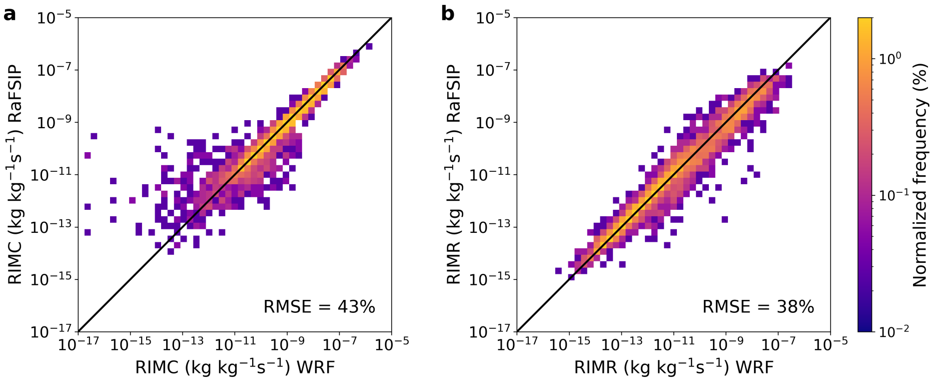

Fig. S8 Normalized histogram of the true WRF results versus the forestrimall predictions used in EC-Earth3-AerChem to predict (**a**) RIMC, and (**b**) RIMR, that are then used as inputs to the RaFSIP scheme. The black line represents the one-to-one line.

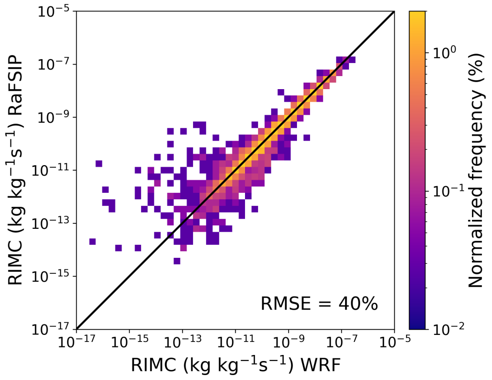

Fig. S9 Normalized histogram of the true WRF results versus the forestrimc predictions used in EC-Earth3-AerChem to predict RIMC, which will then be used as input to the RaFSIP scheme. The black line represents the one-to-one line.

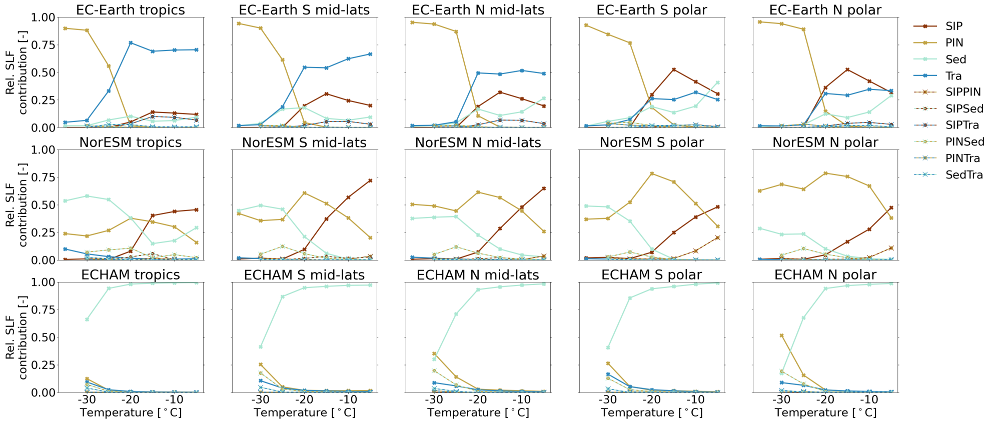

Fig. S10 As Fig. 3 in the main manuscript, but including interactions between all processes. One panel per latitudinal zone.

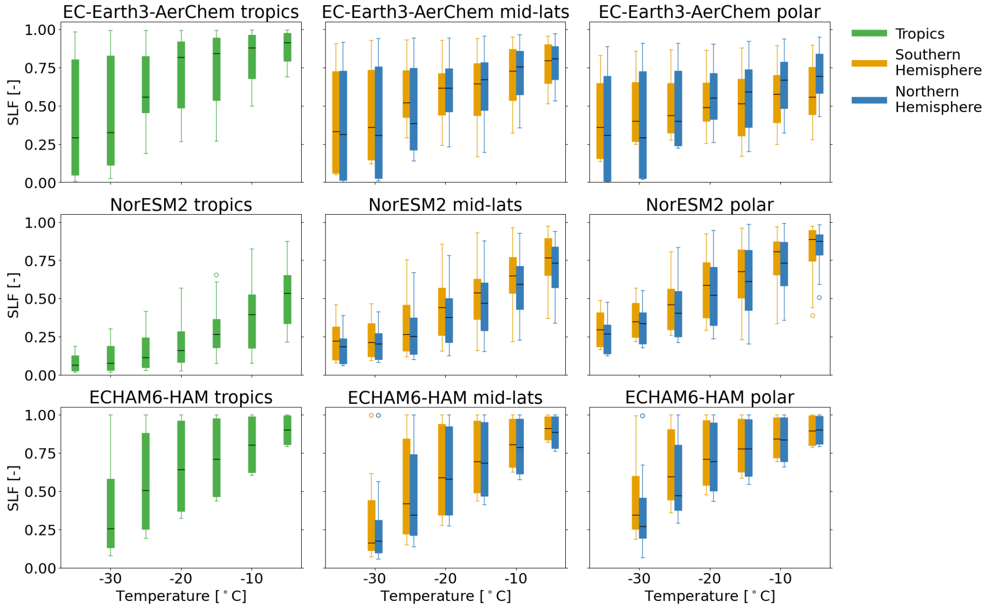

Fig. S11 Boxplots showing the range of SLF annual means in the latitudinal zones between all 16 simulations in (**a-c**) EC-Earth3-AerChem, (**d-f**) NorESM2, (**g-i**) ECHAM6-HAM. Same latitudinal zones as in Fig. 3 in the main manuscript.

Supplementary Tables

Table S1 RMSE of modeled annual zonal SLF compared to GOCCP, as shown in Figs. 1 and S2, averaged over the same latitudinal zones as used in Fig. 3. Prior to averaging, the GOCCP values were interpolated onto the latitudes of the respective model, in contrast to Figs. 1 and S2.

Latitudinal zone	Temperature	EC-Earth3-AerChem	NorESM2	ECHAM6-HAM
S polar	-5° C	0.30	0.15	0.19
S mid-lats	-5° C	0.20	0.06	0.24
Tropics	-5° C	0.40	0.03	0.51
N mid-lats	-5° C	0.13	0.06	0.29
N polar	-5° C	0.20	0.04	0.17
S polar	-10° C	0.32	0.20	0.11
S mid-lats	-10° C	0.23	0.13	0.12
Tropics	-10° C	0.25	0.14	0.35
N mid-lats	-10° C	0.14	0.17	0.14
N polar	-10° C	0.30	0.20	0.05
S polar	-15° C	0.33	0.19	0.09
S mid-lats	-15° C	0.27	0.17	0.05
Tropics	-15° C	0.09	0.10	0.23
N mid-lats	-15° C	0.17	0.18	0.06
N polar	-15° C	0.32	0.22	0.03
S polar	-20° C	0.16	0.13	0.10
S mid-lats	-20° C	0.10	0.14	0.05
Tropics	-20° C	0.08	0.13	0.14
N mid-lats	-20° C	0.05	0.12	0.02
N polar	-20° C	0.13	0.13	0.05
S polar	-25° C	0.09	0.11	0.09
S mid-lats	-25° C	0.04	0.13	0.11
Tropics	-25° C	0.03	0.13	0.04
N mid-lats	-25° C	0.05	0.09	0.05
N polar	-25° C	0.04	0.07	0.03
S polar	-30° C	0.02	0.05	0.08
S mid-lats	-30° C	0.06	0.10	0.13
Tropics	-30° C	0.10	0.10	0.04
N mid-lats	-30° C	0.13	0.05	0.08
N polar	-30° C	0.16	0.01	0.06

Table S1 continued

Latitudinal zone	Temperature	EC-Earth3-AerChem	NorESM2	ECHAM6-HAM
S polar	-35° C	0.04	0.03	0.13
S mid-lats	-35° C	0.05	0.03	0.11
Tropics	-35° C	0.05	0.04	0.04
N mid-lats	-35° C	0.07	0.01	0.07
N polar	-35° C	0.10	0.02	0.11

Table S2 Overview of the switch configuration for each model. CMP: cloud microphysics.

Switch	Affects ice number or mass	EC-Earth3-AerChem	NorESM2	ECHAM6-HAM
Primary ice nucleation (**PIN**)	Number	Switch in CMP routine	Switch in CMP routine	Switch in CMP routine
Secondary ice production (**SIP**)	Number and mass	Switch in CMP routine	Switch in CMP routine	Switch in CMP routine
Sedimentation (**Sed**)	Number and/or mass	Switch in CMP routine (mass only)	Switch in CMP routine (number and mass)	Switch in CMP routine (number and mass)
Transport (**Tra**)	Number and/or mass	Inside CMP: detrainment (mass); outside CMP: horizontal advection, vertical diffusion (mass).	Outside CMP: detrainment, horizontal advection, vertical diffusion (number and mass)	Outside CMP: horizontal advection, vertical diffusion (number and mass).

Table S3 Overview of simulations.

Simulation name	PIN	SIP	Sed	Tra
Ref	✓	✓	✓	✓
NoMPC	✗	✗	✗	✗
NoPIN	✗	✓	✓	✓
OnlyPIN	✓	✗	✗	✗
NoSIP	✓	✗	✓	✓
OnlySIP	✗	✓	✗	✗
NoSed	✓	✓	✗	✓
OnlySed	✗	✗	✓	✗
NoTra	✓	✓	✓	✗
OnlyTra	✗	✗	✗	✓
PINSIP	✓	✓	✗	✗
PINSed	✓	✗	✓	✗
PINTra	✓	✗	✗	✓
SedTra	✗	✗	✓	✓
SIPSed	✗	✓	✓	✗
SIPTra	✗	✓	✗	✓

References

[1] van Noije, T., Bergman, T., Le Sager, P., O'Donnell, D., Makkonen, R., Gonçalves-Ageitos, M., Döscher, R., Fladrich, U., von Hardenberg, J., Keskinen, J.-P., Korhonen, H., Laakso, A., Myriokefalitakis, S., Ollinaho, P., Pérez García-Pando, C., Reerink, T., Schrödner, R., Wyser, K., Yang, S.: EC-Earth3-AerChem: A global climate model with interactive aerosols and atmospheric chemistry participating in CMIP6. Geosci. Model Dev. **14**(9), 5637–5668 (2021) https://doi.org/10.5194/gmd-14-5637-2021

[2] Huijnen, V., Williams, J., van Weele, M., van Noije, T., Krol, M., Dentener, F., Segers, A., Houweling, S., Peters, W., de Laat, J., Boersma, F., Bergamaschi, P., van Velthoven, P., Le Sager, P., Eskes, H., Alkemade, F., Scheele, R., Nédélec, P., Pätz, H.-W.: The global chemistry transport model TM5: Description and evaluation of the tropospheric chemistry version 3.0. Geosci. Model Dev. **3**(2), 445–473 (2010) https://doi.org/10.5194/gmd-3-445-2010

[3] Forbes, R.M., Tompkins, A.M., Untch, A.: A New Prognostic Bulk Microphysics Scheme for the IFS. ECMWF Tech. Memo. 649. ECMWF, Reading, UK (2011). https://doi.org/10.21957/bf6vjvxk

[4] Costa-Surós, M., Gonçalves Ageitos, M., Chatziparaschos, M., Georgakaki, P., Thomas, M.A., Montané Pinto, G., Myriokefalitakis, S., van Noije, T., Le Sager, P., Kanakidou, M., Nenes, A., Pérez García-Pando, C.: Implementation of primary and secondary ice production in EC-earth3-AerChem: Global impacts and insights. EGUsphere **2025**, 1–34 (2025) https://doi.org/10.5194/egusphere-2025-4659

[5] Harrison, A.D., Lever, K., Sanchez-Marroquin, A., Holden, M.A., Whale, T.F., Tarn, M.D., McQuaid, J.B., Murray, B.J.: The ice-nucleating ability of quartz immersed in water and its atmospheric importance compared to K-feldspar. Atmospheric Chem. Phys. **19**(17), 11343–11361 (2019) https://doi.org/10.5194/acp-19-11343-2019

[6] Wilson, T.W., Ladino, L.A., Alpert, P.A., Breckels, M.N., Brooks, I.M., Browse, J., Burrows, S.M., Carslaw, K.S., Huffman, J.A., Judd, C., Kilthau, W.P., Mason, R.H., McFiggans, G., Miller, L.A., Nájera, J.J., Polishchuk, E., Rae, S., Schiller, C.L., Si, M., Temprado, J.V., Whale, T.F., Wong, J.P.S., Wurl, O., Yakobi-Hancock, J.D., Abbatt, J.P.D., Aller, J.Y., Bertram, A.K., Knopf, D.A., Murray, B.J.: A marine biogenic source of atmospheric ice-nucleating particles. Nature

525(7568), 234–238 (2015) https://doi.org/10.1038/nature14986

[7] Abdul-Razzak, H., Ghan, S.J., Rivera-Carpio, C.: A parameterization of aerosol activation: 1. Single aerosol type. J. Geophys. Res. Atmospheres **103**(D6), 6123–6131 (1998) https://doi.org/10.1029/97JD03735

[8] Abdul-Razzak, H., Ghan, S.J.: A parameterization of aerosol activation: 2. Multiple aerosol types. J. Geophys. Res. Atmospheres **105**(D5), 6837–6844 (2000) https://doi.org/10.1029/1999JD901161

[9] Pruppacher, H.R., Klett, J.D.: Microphysics of Clouds and Precipitation, 2. edn. Kluwer Academic, Dordrecht (1997)

[10] Ghan, S.J., Abdul-Razzak, H., Nenes, A., Ming, Y., Liu, X., Ovchinnikov, M., Shipway, B.: Droplet nucleation: Physically-based parameterizations and comparative evaluation. J. Adv. Model. Earth Syst. **3**(4) (2011) https://doi.org/10.1029/2011MS000074

[11] Bechtold, P., Forbes, R., Sandu, I., Land, S., Ahlgrimm, M.: A major moist physics upgrade for the IFS. ECMWF Newsletter **164**, 24–32 (2020)

[12] Forbes, R.M., Geer, A., Lonitz, K., Ahlgrimm, M.: Reducing systematic error in cold-air outbreaks. ECMWF Newsletter **146**, 17–22 (2016)

[13] Seland, Ø., Bentsen, M., Olivié, D., Toniazzo, T., Gjermundsen, A., Graff, L.S., Debernard, J.B., Gupta, A.K., He, Y.-C., Kirkevåg, A., Schwinger, J., Tjiputra, J., Aas, K.S., Bethke, I., Fan, Y., Griesfeller, J., Grini, A., Guo, C., Ilicak, M., Karset, I.H.H., Landgren, O., Liakka, J., Moseid, K.O., Nummelin, A., Spensberger, C., Tang, H., Zhang, Z., Heinze, C., Iversen, T., Schulz, M.: Overview of the Norwegian Earth System Model (NorESM2) and key climate response of CMIP6 DECK, historical, and scenario simulations. Geosci. Model Dev. **13**(12), 6165–6200 (2020) https://doi.org/10.5194/gmd-13-6165-2020

[14] Graff, L.S., Tjiputra, J., Gjermundsen, A., Born, A., Debernard, J.B., Goelzer, H., He, Y.-C., Langebroek, P.M., Nummelin, A., Olivié, D., Seland, Ø., Storelvmo, T., Bentsen, M., Guo, C., Rosendahl, A., Tao, D., Toniazzo, T., Li, C., Outten, S., Schulz, M.: Sensitivity of winter Arctic amplification in NorESM2. EGUsphere, 1–41 (2025) https://doi.org/10.5194/egusphere-2025-472

[15] Kirkevåg, A., Grini, A., Olivié, D., Seland, Ø., Alterskjær, K., Hummel, M., Karset, I.H.H., Lewinschal, A., Liu, X., Makkonen, R., Bethke, I., Griesfeller, J.,

Schulz, M., Iversen, T.: A production-tagged aerosol module for Earth system models, OsloAero5.3 – extensions and updates for CAM5.3-Oslo. Geosci. Model Dev. **11**(10), 3945–3982 (2018) https://doi.org/10.5194/gmd-11-3945-2018

[16] van Marle, M.J.E., Kloster, S., Magi, B.I., Marlon, J.R., Daniau, A.-L., Field, R.D., Arneth, A., Forrest, M., Hantson, S., Kehrwald, N.M., Knorr, W., Lasslop, G., Li, F., Mangeon, S., Yue, C., Kaiser, J.W., van der Werf, G.R.: Historic global biomass burning emissions for CMIP6 (BB4CMIP) based on merging satellite observations with proxies and fire models (1750–2015). Geosci. Model Dev. **10**(9), 3329–3357 (2017) https://doi.org/10.5194/gmd-10-3329-2017

[17] Thomason, L.W., Ernest, N., Millán, L., Rieger, L., Bourassa, A., Vernier, J.-P., Manney, G., Luo, B., Arfeuille, F., Peter, T.: A global space-based stratospheric aerosol climatology: 1979–2016. Earth Syst. Sci. Data **10**(1), 469–492 (2018) https://doi.org/10.5194/essd-10-469-2018

[18] Hurrell, J.W., Hack, J.J., Shea, D., Caron, J.M., Rosinski, J.: A New Sea Surface Temperature and Sea Ice Boundary Dataset for the Community Atmosphere Model. J. Clim. **21**(19), 5145–5153 (2008) https://doi.org/10.1175/2008JCLI2292.1

[19] Gettelman, A., Morrison, H.: Advanced Two-Moment Bulk Microphysics for Global Models. Part I: Off-Line Tests and Comparison with Other Schemes. J. Clim. **28**(3), 1268–1287 (2015) https://doi.org/10.1175/JCLI-D-14-00102.1

[20] Hoose, C., Kristjánsson, J.E., Chen, J.-P., Hazra, A.: A Classical-Theory-Based Parameterization of Heterogeneous Ice Nucleation by Mineral Dust, Soot, and Biological Particles in a Global Climate Model. J. Atmospheric Sci. **67**(8), 2483–2503 (2010) https://doi.org/10.1175/2010JAS3425.1

[21] Wang, Y., Liu, X., Hoose, C., Wang, B.: Different contact angle distributions for heterogeneous ice nucleation in the Community Atmospheric Model version 5. Atmospheric Chem. Phys. **14**(19), 10411–10430 (2014) https://doi.org/10.5194/acp-14-10411-2014

[22] Stevens, B., Giorgetta, M., Esch, M., Mauritsen, T., Crueger, T., Rast, S., Salzmann, M., Schmidt, H., Bader, J., Block, K., Brokopf, R., Fast, I., Kinne, S., Kornblueh, L., Lohmann, U., Pincus, R., Reichler, T., Roeckner, E.: Atmospheric component of the MPI-M Earth System Model: ECHAM6. J. Adv. Model. Earth Syst. **5**(2), 146–172 (2013) https://doi.org/10.1002/jame.20015

[23] Stier, P., Feichter, J., Kinne, S., Kloster, S., Vignati, E., Wilson, J., Ganzeveld, L., Tegen, I., Werner, M., Balkanski, Y., Schulz, M., Boucher, O., Minikin, A., Petzold, A.: The aerosol-climate model ECHAM5-HAM. Atmospheric Chem. Phys. **5**(4), 1125–1156 (2005) https://doi.org/10.5194/acp-5-1125-2005

[24] Zhang, K., O'Donnell, D., Kazil, J., Stier, P., Kinne, S., Lohmann, U., Ferrachat, S., Croft, B., Quaas, J., Wan, H., Rast, S., Feichter, J.: The global aerosol-climate model ECHAM-HAM, version 2: Sensitivity to improvements in process representations. Atmospheric Chem. Phys. **12**(19), 8911–8949 (2012) https://doi.org/10.5194/acp-12-8911-2012

[25] Tegen, I., Neubauer, D., Ferrachat, S., Siegenthaler-Le Drian, C., Bey, I., Schutgens, N., Stier, P., Watson-Parris, D., Stanelle, T., Schmidt, H., Rast, S., Kokkola, H., Schultz, M., Schroeder, S., Daskalakis, N., Barthel, S., Heinold, B., Lohmann, U.: The global aerosol–climate model ECHAM6.3–HAM2.3 – Part 1: Aerosol evaluation. Geosci. Model Dev. **12**(4), 1643–1677 (2019) https://doi.org/10.5194/gmd-12-1643-2019

[26] Proske, U., Ferrachat, S., Neubauer, D., Staab, M., Lohmann, U.: Assessing the potential for simplification in global climate model cloud microphysics. Atmospheric Chem. Phys. **22**(7), 4737–4762 (2022) https://doi.org/10.5194/acp-22-4737-2022

[27] Lohmann, U., Stier, P., Hoose, C., Ferrachat, S., Kloster, S., Roeckner, E., Zhang, J.: Cloud microphysics and aerosol indirect effects in the global climate model ECHAM5-HAM. Atmospheric Chem. Phys. **7**(13), 3425–3446 (2007) https://doi.org/10.5194/acp-7-3425-2007

[28] Lohmann, U., Hoose, C.: Sensitivity studies of different aerosol indirect effects in mixed-phase clouds. Atmospheric Chem. Phys. **9**(22), 8917–8934 (2009) https://doi.org/10.5194/acp-9-8917-2009

[29] Neubauer, D., Ferrachat, S., Siegenthaler-Le Drian, C., Stier, P., Partridge, D.G., Tegen, I., Bey, I., Stanelle, T., Kokkola, H., Lohmann, U.: The global aerosol–climate model ECHAM6.3–HAM2.3 – Part 2: Cloud evaluation, aerosol radiative forcing, and climate sensitivity. Geosci. Model Dev. **12**(8), 3609–3639 (2019) https://doi.org/10.5194/gmd-12-3609-2019

[30] Hoose, C., Lohmann, U., Erdin, R., Tegen, I.: The global influence of dust mineralogical composition on heterogeneous ice nucleation in mixed-phase clouds.

Environ. Res. Lett. **3**(2), 025003 (2008) https://doi.org/10.1088/1748-9326/3/2/025003

[31] Lohmann, U., Diehl, K.: Sensitivity Studies of the Importance of Dust Ice Nuclei for the Indirect Aerosol Effect on Stratiform Mixed-Phase Clouds. J. Atmospheric Sci. **63**(3), 968–982 (2006) https://doi.org/10.1175/JAS3662.1

[32] Stier, P.: Limitations of passive remote sensing to constrain global cloud condensation nuclei. Atmospheric Chem. Phys. **16**(10), 6595–6607 (2016) https://doi.org/10.5194/acp-16-6595-2016

[33] Lohmann, U., Neubauer, D.: The importance of mixed-phase and ice clouds for climate sensitivity in the global aerosol–climate model ECHAM6-HAM2. Atmospheric Chem. Phys. **18**(12), 8807–8828 (2018) https://doi.org/10.5194/acp-18-8807-2018

[34] Korolev, A.: Limitations of the Wegener–Bergeron–Findeisen Mechanism in the Evolution of Mixed-Phase Clouds. J. Atmos. Sci. (2007) https://doi.org/10.1175/JAS4035.1

[35] Phillips, V.T.J., Yano, J.-I., Khain, A.: Ice Multiplication by Breakup in Ice–Ice Collisions. Part I: Theoretical Formulation. J. Atmospheric Sci. **74**(6), 1705–1719 (2017) https://doi.org/10.1175/JAS-D-16-0224.1

[36] Phillips, V.T.J., Patade, S., Gutierrez, J., Bansemer, A.: Secondary Ice Production by Fragmentation of Freezing Drops: Formulation and Theory. J. Atmospheric Sci. **75**(9), 3031–3070 (2018) https://doi.org/10.1175/JAS-D-17-0190.1

[37] Georgakaki, P., Nenes, A.: RaFSIP: Parameterizing Ice Multiplication in Models Using a Machine Learning Approach. J. Adv. Model. Earth Syst. **16**(6), 2023–003923 (2024) https://doi.org/10.1029/2023MS003923

[38] Pedregosa, F., Varoquaux, G., Gramfort, A., Michel, V., Thirion, B., Grisel, O., Blondel, M., Prettenhofer, P., Weiss, R., Dubourg, V., Vanderplas, J., Passos, A., Cournapeau, D.: Scikit-learn: Machine Learning in Python. J. Mach. Learn. Res. **12**, 2825–2830 (2011)

[39] Meyers, M.P., DeMott, P.J., Cotton, W.R.: New Primary Ice-Nucleation Parameterizations in an Explicit Cloud Model. J. Appl. Meteorol. Climatol. **31**(7), 708–721 (1992) https://doi.org/10.1175/1520-0450(1992)031⟨0708:NPINPI⟩2.0.

CO;2
